# Precise Large-Scale Chemical Transformations on Surfaces: Deep Learning Meets Scanning Probe Microscopy with Interpretability

**DOI:** 10.1021/jacs.4c14757

**Published:** 2024-12-16

**Authors:** Nian Wu, Markus Aapro, Joakim S. Jestilä, Robert Drost, Miguel Martínez García, Tomás Torres, Feifei Xiang, Nan Cao, Zhijie He, Giovanni Bottari, Peter Liljeroth, Adam S. Foster

**Affiliations:** † Department of Applied Physics, Aalto University, Helsinki 02150, Finland; ‡ Departamento de Química Orgánica, Universidad Autónoma de Madrid, Madrid 28049, Spain; § IMDEA-Nanociencia, Campus de Cantoblanco, Madrid 28049, Spain; ∥ Institute for Advanced Research in Chemical Sciences, Universidad Autónoma de Madrid, Madrid 28049, Spain; ⊥ nanotech@surfaces Laboratory, Empa-Swiss Federal Laboratories for Materials Science and Technology, Dübendorf 8600, Switzerland; # Department of Computer Science, Aalto University, Helsinki 02150, Finland; ∇ WPI Nano Life Science Institute, Kanazawa University, Kanazawa 610101, Japan

## Abstract

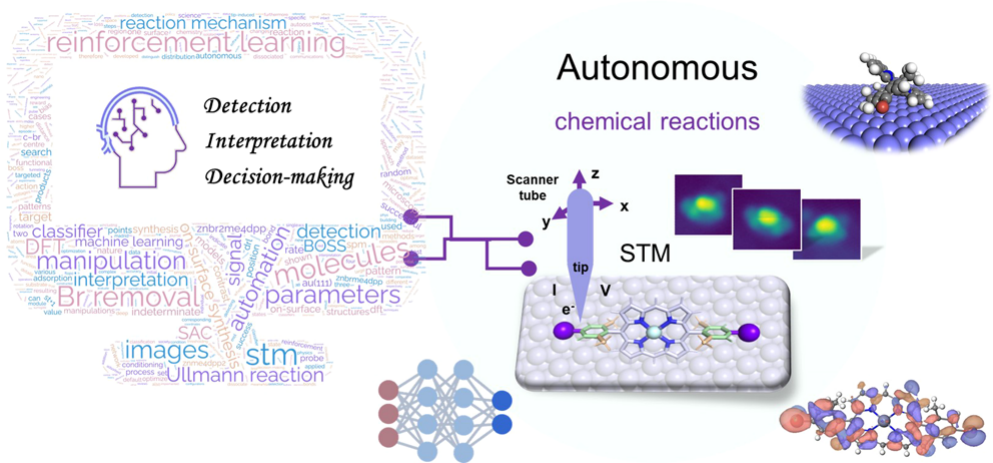

Scanning probe microscopy (SPM) techniques have shown great potential in fabricating nanoscale structures endowed with exotic quantum properties achieved through various manipulations of atoms and molecules. However, precise control requires extensive domain knowledge, which is not necessarily transferable to new systems and cannot be readily extended to large-scale operations. Therefore, efficient and autonomous SPM techniques are needed to learn optimal strategies for new systems, in particular for the challenge of controlling chemical reactions and hence offering a route to precise atomic and molecular construction. In this paper, we developed a software infrastructure named AutoOSS (**Auto**nomous **O**n-**S**urface **S**ynthesis) to automate bromine removal from hundreds of Zn(II)-5,15-bis(4-bromo-2,6-dimethylphenyl)porphyrin (ZnBr_2_Me_4_DPP) on Au(111), using neural network models to interpret STM outputs and deep reinforcement learning models to optimize manipulation parameters. This is further supported by Bayesian optimization structure search (BOSS) and density functional theory (DFT) computations to explore 3D structures and reaction mechanisms based on STM images.

## Introduction

Precisely and controllably manipulating atoms or molecules on surfaces offers the potential for assembling nanomaterials with tunable exotic properties for novel applications in optoelectronics and spintronics.^1−7^ Recently, scanning probing microscopy (SPM), including scanning tunneling microscopy (STM) and atomic force microscopy (AFM), has shown great potential in nanofabrication through complex manipulations including pulling, pushing, pick–transfer–drop, and dissociation.^8−12^ These manipulations are predominantly controlled through the tip position (tip_*x*_, tip_*y*_, tip_*z*_), bias voltage (*V*), and tunneling current (*I*) in STM. However, the selection and optimization of such parameters is a time-consuming and repetitive process and strongly depends on the domain knowledge, which is not necessarily transferable to new systems. Therefore, efficient and autonomous SPM techniques are needed to reduce the reliance on human supervision and efficiently learn optimal strategies for the fabrication of functional nanostructures, particularly to the scale that would have an impact on real technologies.

Advanced machine learning techniques, especially image classification, image segmentation, and reinforcement learning (RL), have recently emerged as promising methods to automate various tasks in SPM, including the identification of optimal sample regions, the evaluation of the quality of scanning images, tip conditioning and the selection of manipulation parameters, and the detection of reaction sites.^13−19^ For example, RL decision-making agents have been developed using discrete actions to find the proper trajectories to lift a large molecule,^20^ and also to laterally manipulate a polar molecule.^21^ In contrast to making decisions within a set of discrete actions, Chen and co-workers developed a deep reinforcement learning (DRL) approach capable of selecting parameters from continuous action space including tip-start and -end positions, bias voltage, and tunneling conductance to steer the motion of atoms.^22^ The advancements in SPM automation mentioned above pave the way for the next step in nanostructure assembly: the automation of chemical reactions.

For the engineering of new organic materials, on-surface synthesis (OSS), which is based on chemical reactions, has developed into a powerful tool for the controllable formation of molecular structures on solid surfaces.^23^ In particular, the ability to control chemical reactions using temperature^24^ and light^25^ in combination with careful selection of molecular precursors has allowed for breakthrough work in the fabrication of carbon nanostructures and organic molecular networks.^26^ Partnered with the high-resolution characterization SPM offers, sequences of on-surface reactions now provide molecular assembly options that are impossible in solution.^27−30^ Alongside this, the concept of single-molecule engineering, to control all of the elementary steps of a molecular chemical reaction via SPM manipulations, was introduced in 2000.^31^ Yet the potential of SPM for single-molecule engineering has only emerged in recent years.^28,32−35^ Despite these exciting results, it is clear that the technical challenges and time demands of manual manipulation approaches are not suitable for fabrication beyond a few molecules, and scaling these procedures beyond single manipulations and reactions to fabricate large molecular assemblies and engineer complex electronic states requires autonomous SPM operation.^30,36^

In this paper, we establish a deep learning workflow to automate STM manipulations and optimize manipulation parameters to efficiently and selectively break C–Br bonds in organobromides. Breaking these bonds is the first step of the Ullmann reaction,^23^ and an important intermediary step in OSS of complex molecules. This is then applied to Zn(II)-5,15-bis(4-bromo-2,6-dimethylphenyl)porphyrin (ZnBr_2_Me_4_DPP) on Au(111) as a model system to study autonomous tip-induced reactions in STM. Meanwhile, density functional theory (DFT)^37^ calculations and Bayesian optimization structure search (BOSS)^38^ serve as auxiliary tools to explore adsorption structures and reaction mechanisms in combination with SPM results and DRL models.

## Results and Discussion

**Figure 1:**
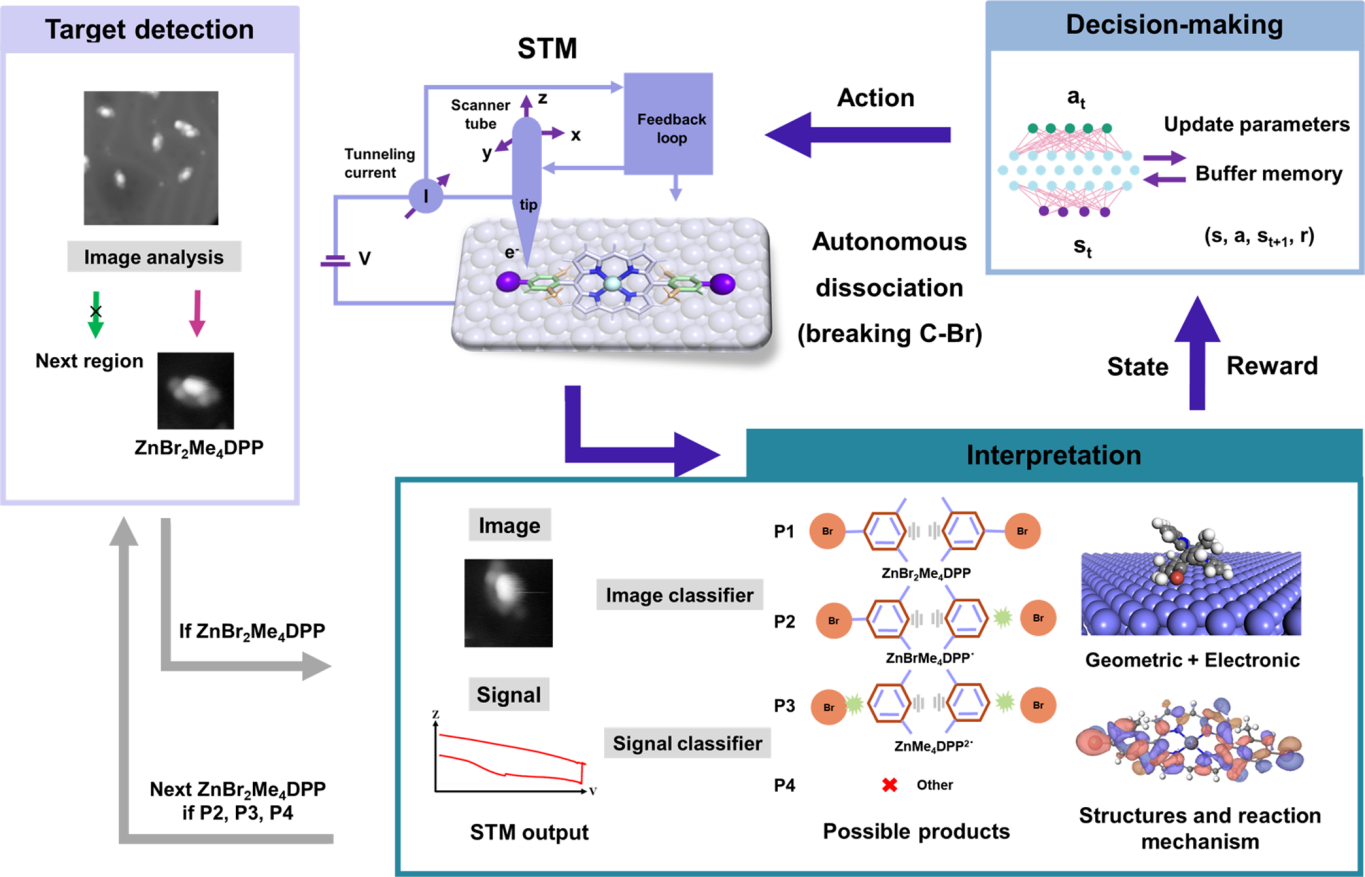
AutoOSS workflow. AutoOSS consists of three key modules: target detection, decision-making, and interpretation. The target detection module is responsible for detecting individual ZnBr_2_Me_4_DPP candidate molecules from a larger scanning image by evaluating distances and areas of image contrast. The interpretation module aims at understanding the effect of manipulation parameters implemented on molecules through identifying products based on STM output (images and signals). The identification of the products determines the next step. The decision-making module generates the manipulation parameters. Here, we primarily employed two methods: a random generator and a DRL approach. The DRL approach searches for optimal STM manipulation parameters toward a goal using a reward system based on the state. Finally, a substantive number of 2D scanning images, reflecting various configurations of molecules on Au(111), collected during the whole process can be used to analyze the geometric and electronic structures and potential reaction mechanisms with BOSS and DFT.

The overall architecture of our software infrastructure **AutoOSS** (**Auto**mated **O**n-**S**urface **S**ynthesis) consists of three components (Figure 1): Target detection, search and identify targeted fragments based on STM images; Interpretation—Models to interpret the STM output during manipulation; Decision-making, DRL agent for selecting SPM parameters.

### Target Detection

**Figure 2:**
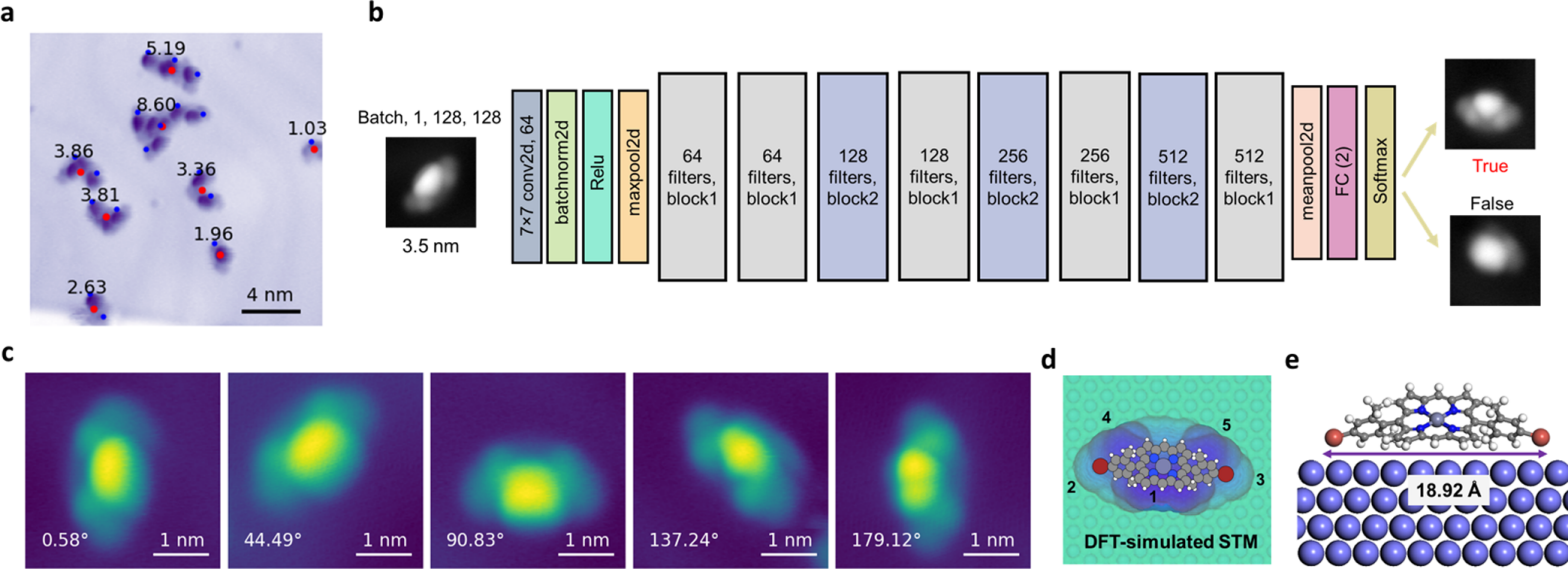
Search and identification of ZnBr_**2**_Me_**4**_DPP. (a) Detection of individual molecules from large images by the distance between the molecules and the area of the contour. Blue points indicate the detected molecules in the contours. Red points represent the center point of contours, whose areas are marked by values. (b) Architecture of the neural network used to predict whether the image includes an individual ZnBr_2_Me_4_DPP. (c) Example of targeted contrast patterns in STM images at different rotation angles. (d) 3D view of DFT simulated STM with a superimposed molecular structure. (e) Side view of ZnBr_2_Me_4_DPP adsorbed on Au(111).

To efficiently detect promising candidate molecules to test C–Br bond dissociation, we acquired an STM image containing several molecules and molecular clusters (see the Methods section for details of the sample preparation). We then analyzed the distance between them (default: 2.5 nm, comparable to the size of molecules) and the area of the associated contrast patterns (default: 1.5–2.5 nm^2^) to exclude clusters or fragmented molecules in Figure 2a. However, many individual fragments share similar areas, especially the dissociated products resulting from the loss of one or two bromine atoms (Figure S9a), which are hard to distinguish from one another. Therefore, we developed a neural network model to identify molecules more precisely based on magnified images focusing on the targeted patterns, where we zoomed in on a smaller scanning region of 3.5 nm × 3.5 nm, still large enough to accommodate the target molecules measuring around 2.3 nm (Figure 2b). Furthermore, the complexity introduced by adsorbing a nonplanar 3D structure onto a 2D surface, where the target molecule can undergo rotations and bind to the substrate at various sites and configurations, inevitably leads to diversity in observed STM contrasts. To understand the features of the molecule for target detection purposes, we correlated the observed STM contrast with multiple configurations aided by simulated STM images (see the Methods section and Figure S5). Among these, we found that the most commonly seen contrast patterns in STM images (four lobes (2, 3, 4, 5) symmetrically around a larger lobe (1) in Figure 2c,d) match well with the three most stable adsorption structures (structures 1, 38, 73, 110, 115, 150, and 158 in Figure S5), which have nearly isoenergetic computed energies ranging from −2.43 to −2.32 eV.

To improve the ability of the models to identify molecules, we defined the most frequently observed molecular features in the STM images as target objects while allowing minor deviations in tip conditions and molecular rotation (Figure 2c). The 3D structure and corresponding STM images (Figure 2d,e) revealed that the central lobe 1 represents the upper periphery of the porphyrin ring; the two lobes of 2 and 3 at the ends are partly due to the presence of Br atoms, and the other two lobes of 4 and 5 originate mainly from the methyl fragments on the phenyl ring. Based on these characteristics, we manually constructed a labeled data set of 1350 images, and an image classifier (Figure 2b) was trained to evaluate whether the scanning image includes an individual ZnBr_2_Me_4_DPP – the ultimate accuracy of the model was 98.5% on the test data set (more details in the Methods section and Figure S12).

### Interpretation

**Figure 3:**
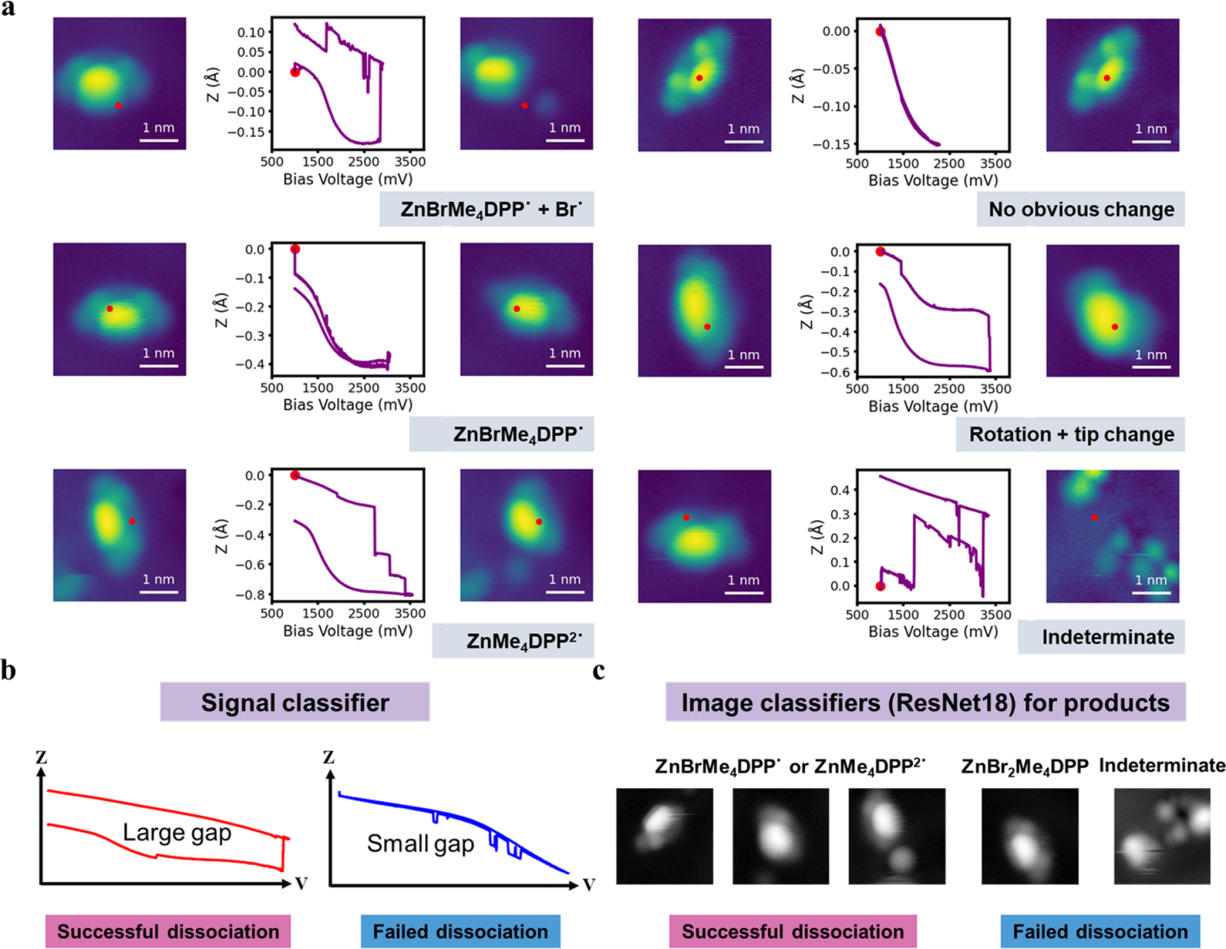
Interpretation of reaction. (a) Possible states after dissociation. The images in the first and third columns refer to STM images before and after dissociation. Red points in the images indicate the tip positions for dissociation. The second column represents the topography change during the implementation of parameters, where the red point marks the initial tip–sample distance. All values are relative to the initial tip–sample distance. (b) Illustration of signal classifier for evaluating whether the dissociation happens. (c) Three categories for evaluating products using image classifiers.

After finding and identifying the target ZnBr_2_Me_4_DPP molecules, we are in a position to initiate the C–Br dissociation process by placing the STM tip on a specific site and applying a voltage bias (ramp pattern or pulse pattern, details shown in Figure S16) and a current. Varying parameters among these four (tip_*x*_, tip_*y*_, *V*, and *I*) may lead to various effects on the molecules, as shown by the representative selection in Figure 3a. The dissociation of the C–Br bond(s), resulting in the corresponding dissociated molecules ZnBrMe_4_DPP^•^ and ZnMe_4_DPP^2•^, is the goal of the manipulation. However, as reflected in STM images, there are multiple possible outcomes of the manipulation process. For example, the contrast pattern of a Br atom (lobe 2 or lobe 3 in Figure 2d) may disappear or coexist near the contrast patterns of ZnBrMe_4_DPP^•^ or ZnMe_4_DPP^2•^. Besides, the appearance and contrast of these patterns may vary due to the possible changes in the STM tip apex during the manipulation process. In addition to changes in the chemical structure of the molecule, some manipulation parameters kept molecules intact, simply resulting in its rotation or translation along the Au(111) surface as well as subtle shape or contrast changes due to different tip conditions. On the other hand, more extreme manipulation parameters can cause destructive damage to molecules and induce breaking of other bonds than C–Br, significant changes such as complete flips of the molecular configuration, large movements of molecules far away from the initial positions, and serious problems in tips like contamination, instabilities, and multiple apexes (see Figure S8).

One of the major challenges for the automation of chemical reactions in SPM is to understand and recognize the consequences of applying manipulation parameters, as outlined in the previous section. Due to the possibility of many complex outcomes, we opted to simply classify all of these into three categories: successful dissociation (Suc), intact molecule (Int), and indeterminate status (Ind), as shown in Figure 3c. This is used to determine if the manipulation action on a targeted molecule has to be continued (in the case of Int) or stopped (in the case of Suc and Ind) and whether the C–Br bond dissociation succeeds (in the case of Suc). For the Int category, the molecule may rotate but retain the typical characteristics of the target molecules, indicating that manipulation can continue. Meanwhile, the Ind status and Suc status mean that the image pattern cannot be characterized as the targeted molecule anymore, and the manipulation process is terminated. The difference between the two is that in the former we cannot establish if the C–Br bond has been dissociated, while in the latter it has clearly succeeded, either resulting in ZnBrMe_4_DPP^•^ or ZnMe_4_DPP^2•^.

Aiming at automating this evaluation process of products, we analyzed over 5000 cases from the STM output (see the Methods section). The most straightforward way is to inspect the images after dissociation. Therefore, we trained classification models (M_Ind_ and M_Diss_) with experts labeling images to predict whether the products are Suc molecules (ZnBrMe_4_DPP^•^ or ZnMe_4_DPP^2•^), ZnBr_2_Me_4_DPP (Int molecules), or belonging to the Ind category (accuracy higher than 97%, more performance matrices and algorithmic details available in Figures S11–14 and the Methods section).

Another obvious signal to consider as a classifier is the bias voltage (*V*)-topography (*Z*) curve; it clearly exhibits different characteristics when resulting in different products during the dissociation (Figure 3b). Generally, successful dissociation tends to be accompanied by a larger hysteresis in their *V*-*Z* curves. While a small hysteresis or even an overlapped curve emerges for manipulation parameters keeping molecules intact, especially those occurring at low voltage or current. Furthermore, we quantitatively estimated the three categories by analyzing the difference in topography between ramping up and down (Diff_topo_) calculated by eq 5 in the section on Signal classification. As shown in Figure S11, there is some overlap in the distribution of the Diff_topo_ for the three categories. However, the values among Int cases are usually smaller than 3.0 nm, and for Suc cases, Diff_topo_ values tend to be larger, even reaching 20 nm, whereas a broader range of Diff_topo_ values (0–63 nm) is observed in Ind cases. While this offers useful insight into the dissociation process in some cases, we found that it was not a reliable classifier for DRL in general, as it was difficult to distinguish among different manipulation effects.

### Decision-Making

#### Random Action

Developing models capable of interpreting STM manipulation outcomes is an essential precondition to finding the optimal parameters to reach the desired goal. Initially, we employed the most straightforward method—random action, to generate the four most relevant manipulation parameters for dissociation of the C–Br bonds: *V*, *I*, tip_*x*_, tip_*y*_.

**Figure 4:**
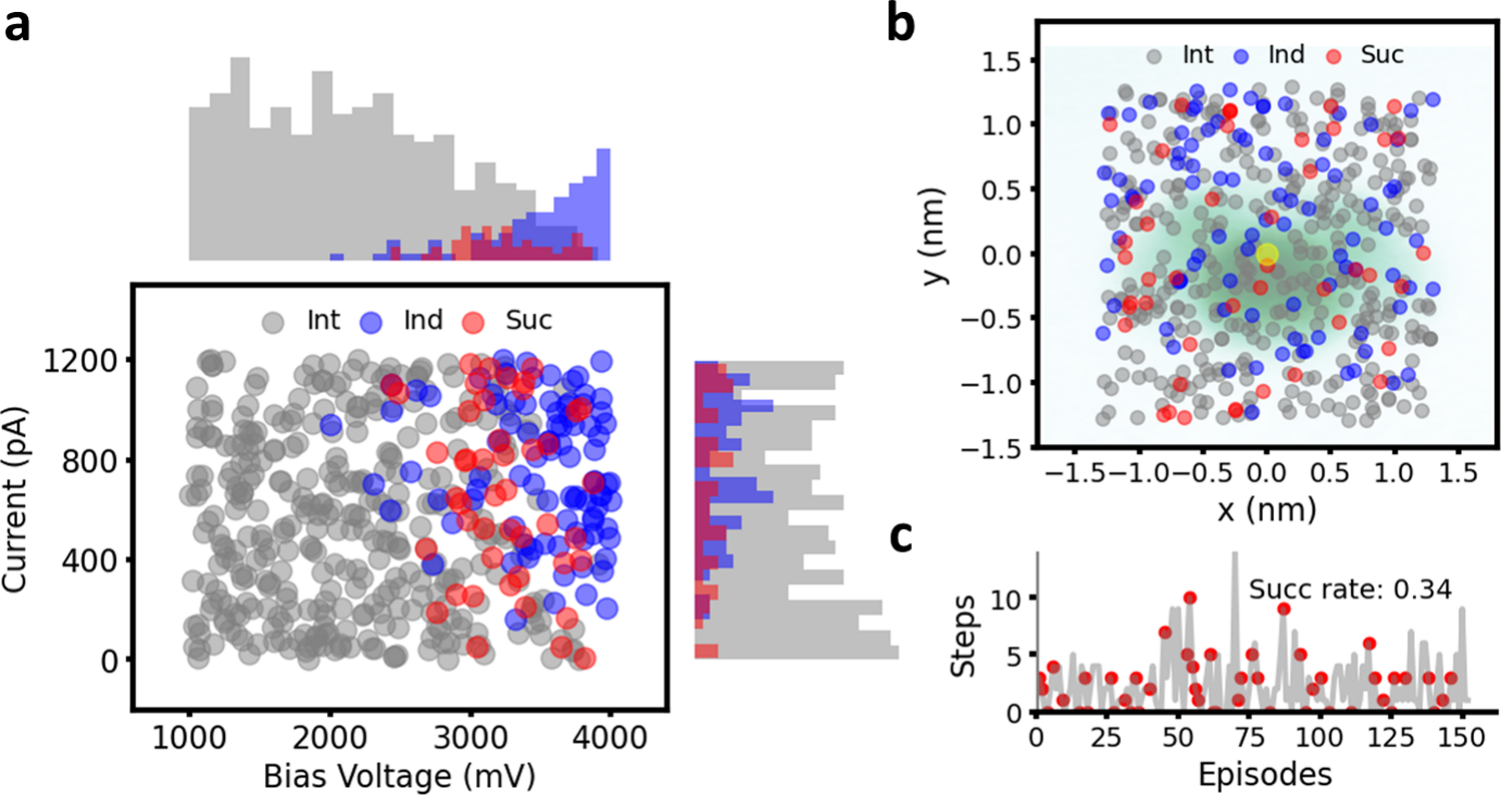
Performance of random action. (a) Distribution of bias voltages and currents during 573 dissociation events. (b) Distribution of tip positions. A ZnBr_2_Me_4_DPP molecule in green is superposed as a reference, where the yellow point represents the center point of the molecule, approximated as an ellipse. (c) Dissociation attempts for each episode before termination, where red points indicate successful dissociation.

We approximate the contrast pattern of a target molecule ZnBr_2_Me_4_DPP as an ellipse, whose center is defined as the reference tip position. Based on the size of the patterns in STM images (about 2.4 nm, Figure S17), we limited the range of possible tip positions to within a radius of 1.3 nm from the reference position, sufficient to cover the whole pattern. In addition, the ranges of voltages and currents are set to 1200–4000 mV and 0–1200 pA based on domain knowledge. Figure 4 demonstrates the effects of 573 dissociation attempts on 150 molecules. Of these, 34% of the molecules (Figure 4c) were successfully dissociated into either ZnBrMe_4_DPP^•^ or ZnMe_4_DPP^2•^, while the majority of molecules were categorized as Ind cases. The voltage and current distribution (Figure 4a) revealed that successful dissociation reactions tend to occur at higher voltages (above 2400 mV), but are also accompanied by a high chance of unwanted reactions. However, the possibility of unwanted reactions could be reduced to some extent by using a lower current. We suspect that a higher current leads to multiple electrons being injected into the molecule, which excites multiple bonds, thus resulting in products that are difficult to analyze. Yet, the dependency of the applied current on the frequency of Ind cases is too noisy to make any clear conclusion in this regard. Meanwhile, lower voltages sometimes result in rotation of the molecule, or no change at all. On the other hand, the dissociation reaction does not seem highly sensitive to the tip position, even when the tip is not directly on top of the molecule, it could still break the C–Br bond as desired. The d*I*/d*V* spectra detected at points 3 and 9 of the molecule (Figure S7) indicate the characteristics of the Au(111) substrate, suggesting that the C–Br bond should be located between point 2 (or 8) and point 3 (or 9), whose distance referred to the center point is less than 0.9 nm (Figure S17). Moreover, we compared the result of the effect of random actions with the tip position constrained to be over molecules by reducing the radius from 1.3 to 0.6 nm in Figure S16a; the success rate slightly increased to 0.39. Meanwhile, the consequence of changing the voltage pattern from a pulse of 8 s to a ramp of 42 s demonstrated a comparable success rate of 0.40 in Figure S16b (more details of the voltage patterns are illustrated in the Methods section, the pulse pattern is the default if not said otherwise).

We further attempted to constrain the tip position near the C–Br bond based on eq 6, which ensures consistent positioning regardless of the rotational state of the contrast patterns in images, and also applied randomly generated bias voltages and currents to dissociate the molecule. The result for 164 molecules in Figure S18 showed a similar trend in the distribution of voltage and current as with random tip position previously used. However, the success rate increased from 0.34 to 0.43, implying that specific tip positions could somewhat reduce the possibility of unwanted reactions of the molecules.

#### Optimize Action by DRL

**Figure 5:**
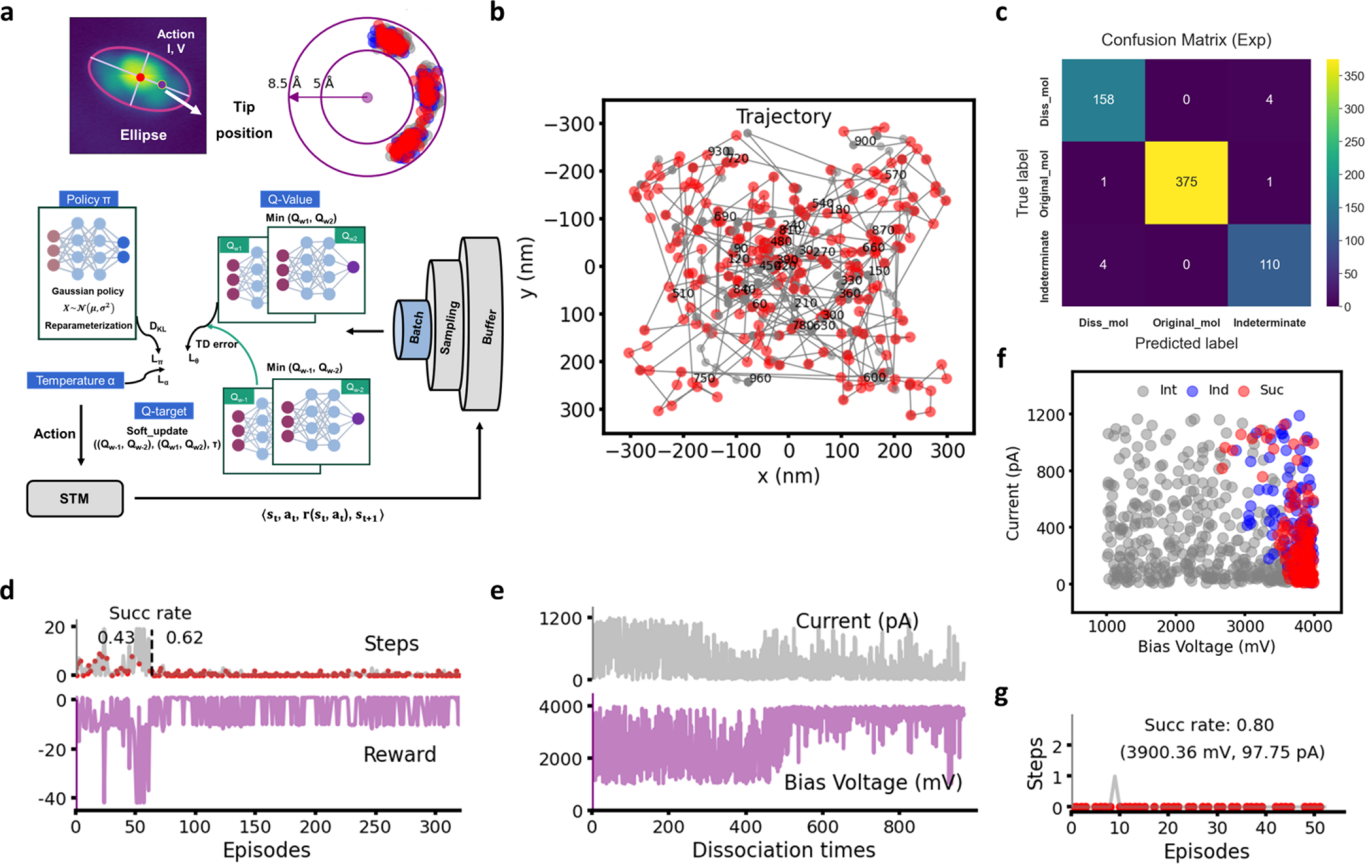
Performance of DRL model. (a) Top: illustration of the fixed tip position referred to the center point of a molecule, approximated as an ellipse (left) and the distribution of all tip positions referred to center points during the DRL training process (right). Bottom: architecture of DRL model based on the SAC algorithm. It consists of a policy network, critic networks (Q-value function), temperature parameter, target Q-Networks, and replay buffer. (b) Real STM trajectory while detecting targeted molecules ZnBr_2_Me_4_DPP on the Au(111) sample and corresponding dissociation results. Red points indicate successful dissociation for the molecules, while gray points represent failed dissociation for the molecules after at most 20 attempts with varying various parameters. Here, xy axes correspond to the STM measurement coordinates. (c) Performance of image classifier on unknown cases. (d) Evolution of dissociate steps (top) and rewards (bottom) over episodes. (e) Evolution of bias voltages (top) and currents (bottom) over dissociation times. (f) Distribution of the pairs of voltage and current implemented on molecules for 968 dissociation times. (g) Repeatability test: dissociate 49 molecules at 3900.36 mV and 97.75 pA.

By definition, the random generator lacks the ability to optimize the dissociation parameters. Generally, this kind of decision-making problem can be formalized as a Markov decision process, where the manipulation parameters (action) depend solely on the current STM image (state). Therefore, we employed a DRL approach based on the Soft Actor-Critic (SAC) algorithm^39^ to optimize parameters for breaking the C–Br covalent bond, using a rational reward design based on interpreting the SPM scanning images during manipulations. To simplify the issue, we hypothesize states in DRL are the same with a 1D state space for all selected ZnBr_2_Me_4_DPP molecules, regardless of tip condition and slight changes in the molecular conformations on the surface. The goal in our DRL models is to optimize the bias voltage and current at the same specific tip position (Figure 5a) under the reward system in eq 7.

Figure 5b displays the trajectory of 328 episodes with a total of 968 dissociation manipulations on the ZnBr_2_Me_4_DPP molecules. The *xy* coordinates correspond to the real coordinates in the STM, reflecting the distribution of the molecules in this region. The red points indicate molecules that underwent successful dissociation, while the gray points indicate indeterminate cases. Note that the heterogeneity in success rate across the surface is a function of nonuniform distribution of molecules on the surface, interruptions in scanning for technical reasons and also the influence of regions used for tip conditioning, and it is difficult to make any inferences on the role of the surface itself. The confusion matrix in Figure 5c further confirms the high accuracy of image classifier models on the unknown data set. Figure 5d illustrates that the model starts to converge after 60 episodes, with a success rate before 60 episodes of 0.43, consistent with that in the tests using random voltage and current. After 60 episodes, the success rate increases to 0.62, and the dissociation steps per episode are fewer than 3 in most cases, also with a higher occurrence of larger accumulated rewards. The fluctuation of rewards between 1 and −10 could be attributed to the high proximity between parameters leading to successful and indeterminate dissociation. Due to differences in molecular conformations on Au(111) and tip conditions, parameters that lead to successful C–Br dissociation for one molecule may result in an indeterminate dissociation for another. This is confirmed by repeatedly testing these successful parameters to dissociate molecules (Figure S19), where the success rate is just 0.42, comparable to that in random dissociation. Meanwhile, the voltage converged to higher values (more than 3500 mV), whereas the current narrowed to lower values (less than 400 pA), despite some fluctuations, as shown in Figure 5e,f. Such narrowing of the range of voltage and current guided by the reward decreases the dissociation steps per episode and increases the success rate. Furthermore, the analysis for the distribution of these parameters in Figure S20 implies that the sets of parameters with voltage higher than 3800 mV and current less than 200 pA offer a higher possibility to successfully dissociate molecules and reduce indeterminate cases.

To explore whether we can further increase the success rate, we randomly selected a set of parameters with a lower current value (97.75 pA) and higher voltage value (3900.36 mV) from those associated with a high likelihood of successful dissociation in DRL training and repeatedly applied these parameters to 49 molecules, as shown in Figure 5g, obtaining an increase of the success rate up to 0.8. This demonstrates the feasibility of our model applied to long-term, selective, efficient operations for autonomous on-surface synthesis in STM. Furthermore, the orbital energies of the highly localized C–Br σ* states of the adsorbed ZnBr_2_Me_4_DPP molecule with respect to the Fermi level at 3.7 eV (Figure S10e), comparable to the voltage bias applied to promote successful dissociation, suggest that selective bond dissociation is probably achieved by tunneling electrons into the corresponding antibonding states, consistent with the literature.^32,40,41^

## Conclusions

To summarize, we have demonstrated the capability of a deep learning model to identify reactants and products based on STM outputs, enabling a DRL agent to evaluate various manipulation parameters. Furthermore, the establishment of a deep reinforcement learning approach allows the agent to optimize these parameters. These advancements address key challenges in STM automation and molecular synthesis. Ultimately, the integration of target detection module, interpretation module, and decision-making module into the AutoOSS workflow achieved the automation of tip-induced C–Br bond breaking from ZnBr_2_Me_4_DPP in STM. AutoOSS enables long-term, selective, and efficient operations without human intervention. Moreover, the extensive data set accumulated from experiments, combined with big-data analysis, DFT calculations, and BOSS, offers the opportunity to uncover hidden physical information, explore 3D molecular conformations, and investigate reaction mechanisms despite the limits of resolution in STM images.

AutoOSS paves the way for automating manipulations in on-surface synthesis, thus, pioneering a new paradigm in single-molecular engineering. Moving forward, we anticipate the possibility of extending AutoOSS to a diverse array of molecules and applications pertinent to complex chemical reactions, encompassing various chemical bonds, molecules, tips, and manipulation types. For similar reaction processes, it could be flexibly transferred to different molecule and substrate combinations through retraining the model with appropriate classifiers of reaction success. Furthermore, there is the potential to enhance the model’s selectivity and precision by using a refined tip, optimized bias voltage pattern, or incorporating AFM signals into the workflow to provide atom-level resolution scanning images.

## Methods

### Experimental Preparation and STM Microscopy

**Figure 6:**
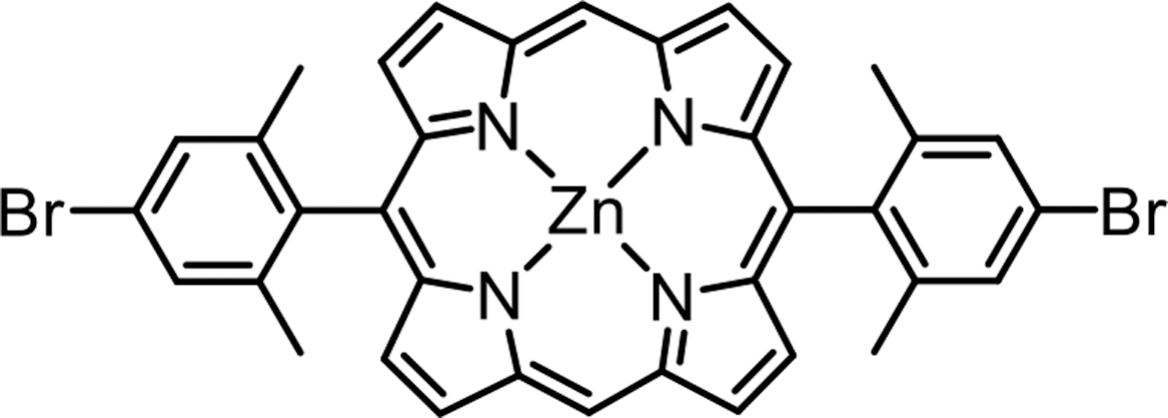
Chemical structure of ZnBr_2_Me_4_DPP.

ZnBr_2_Me_4_DPP molecules (chemical structure shown in Figure 6) were synthesized via the precursors 5,15-bis(4-bromo-2,6-dimethylphenyl)porphyrin (H_2_Br_2_Me_4_DPP) from 2,6-dimethyl-4-bromobenzaldehyde, as shown in Figure S22. Characterizations associated with ZnBr_2_Me_4_DPP molecules and precursors H_2_Br_2_Me_4_DPP (Figures S23–S30), including ^1^H and ^13^C nuclear magnetic resonance (^1^H NMR and ^13^C NMR) spectroscopy, mass spectrometry (MS), and ultraviolet–visible (UV/vis) spectroscopy, were implemented. Then, ZnBr_2_Me_4_DPP molecules were evaporated from a Knudsen cell heated to 230 °C onto a Au(111) sample kept below the 7 K temperature. The STM scanning and dissociation manipulations were performed in constant current mode on a Createc LT-STM system with a gold-coated PtIr tip. The STM images recorded at different scales from 100 to 3.5 nm are shown in Figure S1. Contrast-adjusted STM images in Figure S3 show examples of different adsorption sites of individual molecules on the Au(111) surface. Ultimately, we chose 20 nm × 20 nm to detect promising targeted molecules and 3.5 nm × 3.5 nm to make further identification by neural networks and dissociation manipulations. The scanning speed and the number of pixels for all images are 1000 Å/s and 128, resulting in around 42 s per image.

### Spiral Path Planning

The approach area was about 700 nm × 700 nm, where four 100 nm × 100 nm squares near the boundary were set aside to form tips. Therefore, the manipulation region usually corresponded to the XY coordinates in STM from −300 to 300 nm, where the center point of the region of 20 nm × 20 nm for detecting target candidates was updated by the shortest distance *d*_dist_ away from the reference point beyond the forbidden area. It was formulated as *d*_dist_ = *d*_Eucli_ + α*d_Manha_, where *d*_Eucli_ and *d*_Manha_ indicate Euclidean distance  and Manhattan distance *d*_Manha_ = |*x* – *x*_ref_| + |*y* – *y*_ref_|, respectively, and the coefficient of α is set as 1.

### Detect Target Candidates

We first converted raw scanning images from STM to grayscale images and then made further analyses to detect target candidates using two methods. One method is to limit the distance between image contrast patterns, where binary images with threshold pixel values of 50 (pixels less than 50 were set to 0) and 150 (pixels greater than 150 were set to 255) were obtained to find the individual molecules through a thresholding distance (default: 2.5 nm) between points to get rid of dimer, trimer, or clusters. Another method is to limit the area of patterns, for which we detected contours by the Otsu algorithm^42,43^ with a clear outline (other two algorithms—global thresholding and Otsu thresholding after Gaussian filtering were compared by corresponding areas in Figure S2). Based on the statistical analysis of candidates, we restricted the area of patterns within 1.5–3.0 nm^2^ to further exclude some individual fragments.

### BOSS

We employed the BOSS method^38^ to reduce the number of DFT evaluations needed to map out the configurational phase space. Data points were initialized with a quasi-random Sobol sequence, and the GP-Lower Confidence Bound acquisition function with increasing exploration (elcb) was used on all runs. The kernels for rotation and *xy*-translation were standard periodic kernel (stdp), while the *z*-coordinate used radial basis functions (rbf). The surface symmetry was exploited to multiply the acquired data points by applying symmetry operations to the adsorbate at high-symmetry sites, where the Au(111) surface has three rotationally equivalent sites in addition to two translationally equivalent ones. Initially, a conformational search was conducted on the isolated gas-phase ZnBr_2_Me_4_DPP molecule, with the search variables being full rotation of the phenyl moieties and their methyl substituents (6D search). The surrogate model was constructed from 407 DFT data points. The search resulted in one single main conformer in terms of phenyl rotation, as shown in Figure S4, which was subsequently employed as the molecular building block in the adsorption structure search. The same structure was used as the building block for ZnBrMe_4_DPP^•^ and ZnMe_4_DPP^2•^, since loss of the terminal Br atoms does not result in significant rearrangement of the rest of the molecule following DFT relaxation. The adsorption structure search was done by constructing a surrogate model of the DFT (PBE+vdW^surf^) PES for the translational and rotational degrees of freedom (6D search) and subsequently relaxing the lowest-energy surrogate model local minima with DFT, thus accounting for any changes in the structures of the isolated rigid molecular building blocks enabled by the surface interaction. The molecular adsorbate building blocks for the search were the lowest-energy ZnBr_2_Me_4_DPP, ZnBrMe_4_DPP^•^, and ZnMe_4_DPP^2•^ species as described above, which were combined with the relaxed 11 × 12 × 4 Au(111) substrate building block. The surrogate models for the adsorption structures were constructed out of 262, 94, and 108 data entries for ZnBr_2_Me_4_DPP, ZnBrMe_4_DPP^•^, and ZnMe_4_DPP^2•^, respectively. The global minimum predictions oscillated between the symmetrically equivalent rotational configurations (±60°).

### DFT Calculations

All DFT computations were performed using FHI-aims.^37^ For the initial conformational search using BOSS, we employed the B3LYP functional^44,45^ with light defaults and first-tier basis functions. Subsequently, the resulting global minimum conformers, substrate, and all adsorption structures were relaxed to a force less than 0.01 eV/Å^2^ using the Perdew–Burke–Ernzerhof (PBE) functional augmented with the van der Waals dispersion correction, including collective screening effects of the substrate electrons (vdW^surf^), fully denoted (PBE+vdW^surf^).^46,47^ This choice of functional for both isolated and adsorbed molecules was motivated by the properties of an adsorbed configuration being of interest, for which this functional has been demonstrated accurate in comparison with experiments.^48^ The same functional was also used during BOSS data acquisition iterations for the adsorption structures. The Brillouin zone was sampled using a 1 × 1 × 1 Monkhorst–Pack grid, and the slab was constructed using four layers of 11 × 12 gold atoms as the Au(111) surface, of which the two lowest layers were kept fixed during all computations. The length of the box in the *z*-direction was in total 60 Å, ensuring sufficient vacuum space. This relatively large slab size was chosen to avoid interactions with the adjacent adsorbates. Spin polarization was used for the dissociated ZnBrMe_4_DPP^•^ and ZnMe_4_DPP^2•^ species.

The STM images were simulated using FHI-aims with the Tersoff-Hamann approximation as implemented therein.^49^ The simulation bias was kept at 1.0 V for all images, which were created with VESTA^50^ using an isovalue between 10^–10^ and 10^–12^ a.u. to match experimental STM images.

The calculations for the C–Br dissociation model reactions were performed using the climbing image nudged elastic band and growing strings methods^51,52^ as implemented in https://gitlab.com/cest-group/aimsChain-py3. The pathways of both bond cleavage reactions were modeled using 12 images in total, where the growing string force threshold was 0.5 eV/Å^2^, while the climbing image threshold was 0.05 eV/Å^2^. The initial structure for the reaction was the global minimum adsorption configuration as determined by BOSS, while the dissociated final structures of each step in the reaction were determined by moving the Br atom 5 Å away from the rest of the porphyrin, and relaxing with DFT to the force threshold as the initial image.

The adsorption energy is formulated as *E*_ads_ = *E*_mol+sub_ – *E*_mol_ – *E*_sub_, where mol+sub denotes molecule on the substrate, mol is the isolated molecule, and sub is the isolated substrate.

### Image Classification

All image classifiers were developed based on the ResNet18 model^53^ (the architecture of the neural network shown in Figures 2b and S6), taking STM images with a size of 3.5 nm × 3.5 nm and the pixel numbers of 128 as input. To ensure the intact pattern of fragment in the image, we adjust the scanning region based on the center of the pattern in STM images and scan again if the center point is beyond the threshold region. The default criterion for the center in the pattern is less than 0.438 nm along both *xy* axes, referred to as the center point of the scanning region.

The image classifiers consist of three binary models (M_Target_, M_Ind_, M_Diss_) and one multiclass model (M_Triple_) with the numbers of corresponding data sets shown in Table 1, intended for detecting reactants and distinguishing products. Due to the complexity in products, caused by variable tip conditions, various conformations, and subtle differences for dissociated molecules and pristine molecules, we trained another two binary models (M_Ind_ and M_Diss_) for more elaborate distinctions to supplement M_Triple_. In brief, these models were designed to distinguish ZnBr_2_Me_4_DPP and non-ZnBr_2_Me_4_DPP (M_Target_), to distinguish indeterminate and non-indeterminate (M_Ind_) and to distinguish intact molecules (ZnBr_2_Me_4_DPP) and dissociated molecules (ZnBrMe_4_DPP^•^ or ZnMe_4_DPP^2•^) (M_Diss_).

**Table 1:** Dataset for Four Images Classifiers

target or not			indeterminate or not		
class	train	test	class	train	test
true mol	273	39	indeterminate	1186	270
non-true	1116	159	non-indeterminate	2764	607

dissociation or not			products		
class	train	test	class	train	test
original mol	2023	438	original mol	2023	438
diss mol	741	169	diss mol	741	169
			indeterminate	1186	270

The Adam optimizer^54^ with cross-entropy loss function and StepLR were used to optimize parameters in models. In addition, Bayesian optimization was introduced to optimize the learning rate based on the converged loss values under corresponding learning rate values. Eventually, the optimal learning rates in Adam optimizer are 1.11 × 10^–5^, 4.22 × 10^–5^, 5.84 × 10^–5^, and 0.0001 for M_Target_, M_Ind_, M_Diss_, and M_Triple_, respectively. All models perform decently with accuracy more than 94% and the area under the curve (AUC) higher than 98% (more performance metrics are shown in Figures S12–S15 and Table S4). A confusion matrix divides the classification results into 4 categories through comparing the true values and predicted values: True Position (TP, both real values and predicted values are 1), True Negative (TN, both real values and predicted values are 0), False Positive (FP, real values are 0, but predicted values are 1), and False Negative (FN, real values are 1, but predicted values are 0).
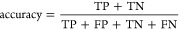
1
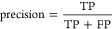
2
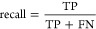
3
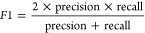
4

### Signal Classification

We tested two types of voltage bias patterns—a ramp of 42 s and a pulse of 8 s, with similar processes, as shown in Figure S16. Times were divided into 1024 steps. The initial voltages are 1 V: for a ramp pattern, the voltage starts to increase to the specific voltages from point 20 until point 512, symmetrically, then decrease to 1 V at point 1004; while for a pulse pattern, the voltage directly jumps to the specific voltage at point 20, which is maintained until point 1004, then back to 1 V. We analyzed signal changes during the dissociation by the difference of topography, formulated as follows
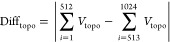
5where *V*_topo_ indicates the value of topography at a point during the process of voltage variation along 1024 points.

### Specific Tip Position


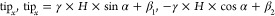
6where *H* and α are the height and the angle of an ellipse evaluated by the fitEllipse function in OpenCV,^55^ γ is a coefficient (default: 0.3), and β_1_ and β_2_ are random noise ranging from −0.1 to 0.1 Å.

### Reward Design

The assessments from image classifiers are applied to evaluate the reward and make further decisions. The reward is defined as
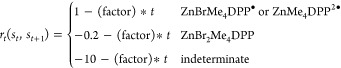
7where *t* indicates the dissociate times in an episode and factor is a coefficient with a default value of 0.2.

### Success Rate

The success rate in a test is defined as
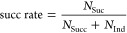
8In this equation, *N*_Suc_ and *N*_Ind_ represent, respectively, the number of successful dissociations and the number of indeterminate cases for all episodes in one test. The value is used to evaluate the ability of the model optimizing parameters to avoid indeterminate cases. Dissociation steps in an episode can be used to assess how fast the model can find successful dissociation parameters. Therefore, the unchanged dissociation is not necessarily considered here.

### Tip Conditioning

The tip may suffer bluntness, contamination, instability, damage, or multiple tips during the scanning process. Correspondingly, different parameters related to voltage, indentation depth, and time may be needed to identify the condition for a sharp and stable tip. To maintain a good tip, the workflow monitors the tip condition and reforms when needed. Experience in this task demonstrates that random approach heights ranging from 2 to 5.5 nm and constant voltage of 1 V usually can achieve a decent tip. As the criteria for successful tip conditioning, we search for candidate molecules in a 20 nm × 20 nm image; if this fails many times (default: 4), our algorithm tries deeper immersion with 10 nm. This strategy allows long-term operations in the whole workflow ranging from detection to dissociation. Tip conditioning is activated only when detecting molecules. Once the targeted molecule is found, it consecutively tests different dissociation parameters until it terminates, without interruption from the conditioning tip. On the one hand, the movement during the tip conditioning may lead to the shift of coordinates in the STM and the tip status after forming may be complex and still effectively bad, which may damage targeted molecules. On the other hand, if the molecules are damaged by a dissociation manipulation rather than tip itself, we treat it as a failed manipulation and classify it as an indeterminate case. Furthermore, empirically, gentle manipulations during dissociation in our task sometimes even make the tip better in obtaining high-quality scanning images. Therefore, tip conditioning during the dissociation attempts is not necessary.

To avoid moving the tip long distances, four square regions with a length of 100 nm near the edge of the approach area were set, among which the tip moves toward the closest one for conditioning. To reduce the time on scanning the tip conditioning region, we just condition the tip at a random point in the square in practice. As an option, the algorithm of detecting point from the blank region to avoid molecules (Figure S21) is available.

### Soft Actor-Critic

The SAC approach consists of a policy sampling module for mapping a state to an action, two state-value q networks for evaluating the state-value, and one state-action value q network. The maximum entropy RL in the model maximizes the cumulative rewards and also pursues the diversity of policy through introducing the entropy term
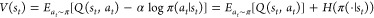
9
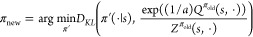
101. Value network: The loss function of value:
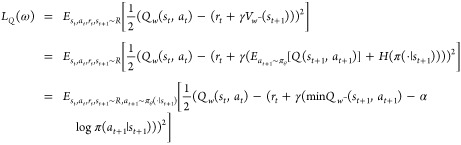
112. Policy network: The action was determined by a policy network, which generated the mean and std of Gaussian distribution by 1 linear layer (hidden_dim: 512) and then sampled action from the Gaussian distribution. The loss function of policy was set based on the Kullback–Leibler (KL) Divergence:^56^
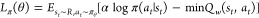
123. Entropy regularization: To maximize the entropy, the corresponding loss function was set as follows:
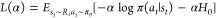
13where α is the temperature parameter. In addition, the advanced sampling technique, Hindsight Experience Replay (HER),^57^ was used to improve the data efficiency. The optimal hyperparameters found in testing are learning rate lr of 0.0003, discount factor γ of 0.99, and target smoothing pf τ of 0.1.

## Data Availability

A video demonstrating AutoOSS’s ability to autonomously and selectively control the reaction, all training data set and parameters in machine learning models, and input and output of BOSS and DFT calculations can be obtained on the Zenodo repository at 10.5281/zenodo.13761822. The source codes and examples are available on the GitHub repository at https://github.com/SINGROUP/AutoOSS.

## Supporting Information Available

Detection of individual ZnBr_2_Me_4_DPP: STM images at different scales, measuring the area of contrast patterns, individual molecules on Au(111), conformational analysis of ZnBr_2_Me_4_DPP, various configurations of ZnBr_2_Me_4_DPP on Au(111), ResNet18 Block, d*I*/d*V* spectra and maps of ZnBr_2_Me_4_DPP on Au(111); interpretation: example of indeterminate cases, properties of contrast patterns in STM images, reaction energies and possible dissociation mechanisms, signal classifier, performance metrics of M_Target_, performance metrics of M_Triple_, performance metrics of M_Ind_, performance metrics of M_Diss_; decision-making: random action at different bias patterns, contrast pattern measurement, random action with fixed tip position, random selection from successful dissociation parameters, dissociation parameters from the DRL model, tip conditioning region; synthesis and characterization: materials and methods, synthesis of precursors H_2_Br_2_Me_4_DPP and ZnBr_2_Me_4_DPP, as well as their corresponding ^1^H NMR, ^13^C NMR, MS, and UV/vis (PDF)
AutoOSS’s ability to autonomously and selectively control the reaction (MP4)
