# Supplementary material for: Precise Large-Scale Chemical Transformations on Surfaces: Deep Learning Meets Scanning Probe Microscopy with Interpretability
Source: J Am Chem Soc. 2024 Dec 16;147(1):1240–50. doi: 10.1021/jacs.4c14757 (PMC11726549; doi:10.1021/jacs.4c14757)
Supplement: Supplementary file 1 — ja4c14757_si_001.pdf [file ja4c14757_si_001.pdf]

Supplementary Information for: Precise large-scale chemical transformations on surfaces: deep learning meets scanning probe microscopy with interpretability

Nian Wu,[*,†] Markus Aapro,[†] Joakim S. Jestilä,[†] Robert Drost,[†] Miguel Martínez García,[‡,¶] Tomás Torres,[‡,§,¶] Feifei Xiang,[‖] Nan Cao,[†] Zhijie He,[⊥] Giovanni Bottari,[‡,§,¶] Peter Liljeroth,[*,†] and Adam S. Foster[*,†,#]

†*Department of Applied Physics, Aalto University, Helsinki, 02150, Finland.*

‡*Departamento de Química Orgánica, Universidad Autónoma de Madrid, Madrid, 28049, Spain*

¶*IMDEA-Nanociencia, Campus de Cantoblanco, Madrid, 28049, Spain*

§*Institute for Advanced Research in Chemical Sciences, Universidad Autónoma de Madrid, Madrid, 28049, Spain*

‖*nanotech@surfaces Laboratory, Empa-Swiss Federal Laboratories for Materials Science and Technology, Dübendorf, 8600, Switzerland*

⊥*Department of Computer Science, Aalto University, Helsinki, 02150, Finland.*

#*WPI Nano Life Science Institute, Kanazawa University, Kanazawa, 610101, Japan.*

E-mail: nian.wu@aalto.fi; peter.liljeroth@aalto.fi; adam.foster@aalto.fi

List of abbreviations

AcOEt = Ethyl acetate

AFM = Atomic Force Microscopy

AUC = Area under the curve

BOSS = Bayesian Optimization Structure Search

CREST = Conformer-Rotamer Ensemble Sampling Tool

DFT = Density Functional Theory

DCM = Dichloromethane

DCTB = trans-2-[3-(4-tert-butylphenyl)-2-methyl-2-propenylidene]malononitrile

DDQ = 2,3-Dichloro-5,6-dicyano-1,4-benzoquinone

DRL = Deep reinforcement learning

HER = Hindsight Experience Replay

MALDI-TOF = Matrix-assisted laser desorption/ionization-time of flight

MS = Mass spectrometry

NMR = Nuclear magnetic resonance

OSS = On-surface synthesis

Rf = Retention factor

RL = Reinforcement learning

SAC = Soft Actor-Critic

SPM = Scanning Probe Microscopy

STM = Scanning Tunnelling Microscopy

THF = Tetrahydrofurane

UV/vis = Ultraviolet-visible

$H_2Br_2Me_4DPP$ = 5,15-bis(4-bromo-2,6-dimethylphenyl)porphyrin

$ZnBr_2Me_4DPP$ = Zn(II)-5,15-bis(4-bromo-2,6-dimethylphenyl)porphyrin

Detection of individual $ZnBr_2Me_4DPP$

STM images at different scales

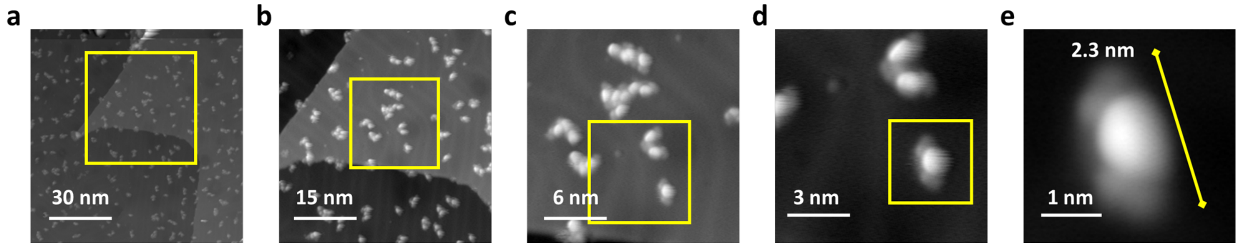

Figure S1: **STM images at difference scales.** (a) 100 nm × 100 nm, (b) 50 nm × 50 nm, (c) 20 nm × 20 nm, (d) 10 nm × 10 nm, (e) 3.5 nm × 3.5 nm.

s

Measuring the area of contrast patterns

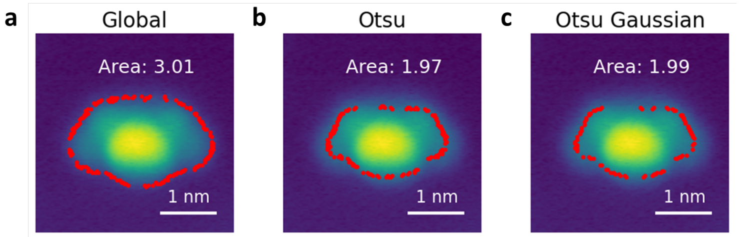

Figure S2: **Comparison of three image thresholding methods.** (a) Global, (b) Otsu and (c) Otsu Gaussian for detecting the contrast pattern in an STM image.

Individual molecules on Au(111)

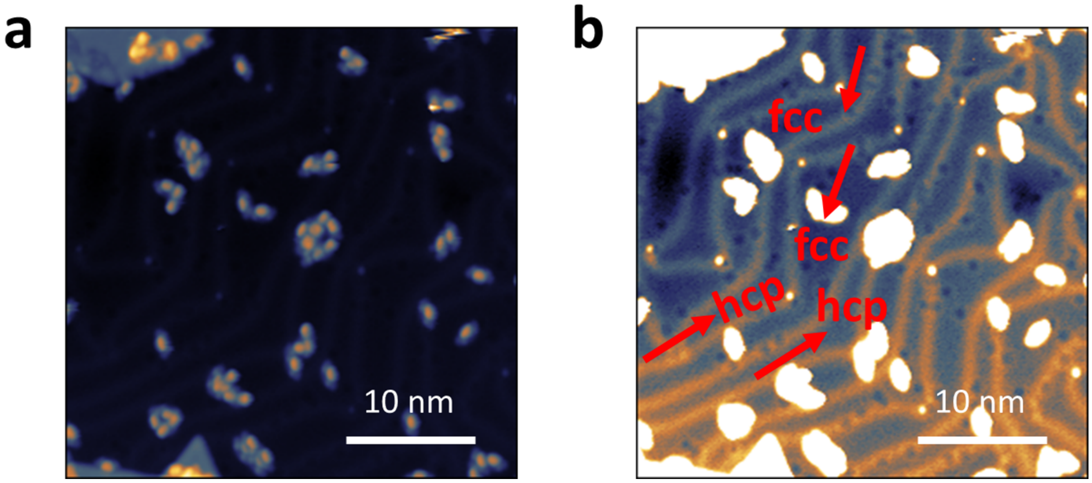

Figure S3: (a, b) STM image of target molecules adsorbed at the hexagonal close-packed (hcp), face-centered cubic (fcc) and elbow regions of the reconstructed Au(111) surface.[1] In (b), the herringbones are visible by adjusting the image contrast.

Conformational analysis of $ZnBr_2Me_4DPP$

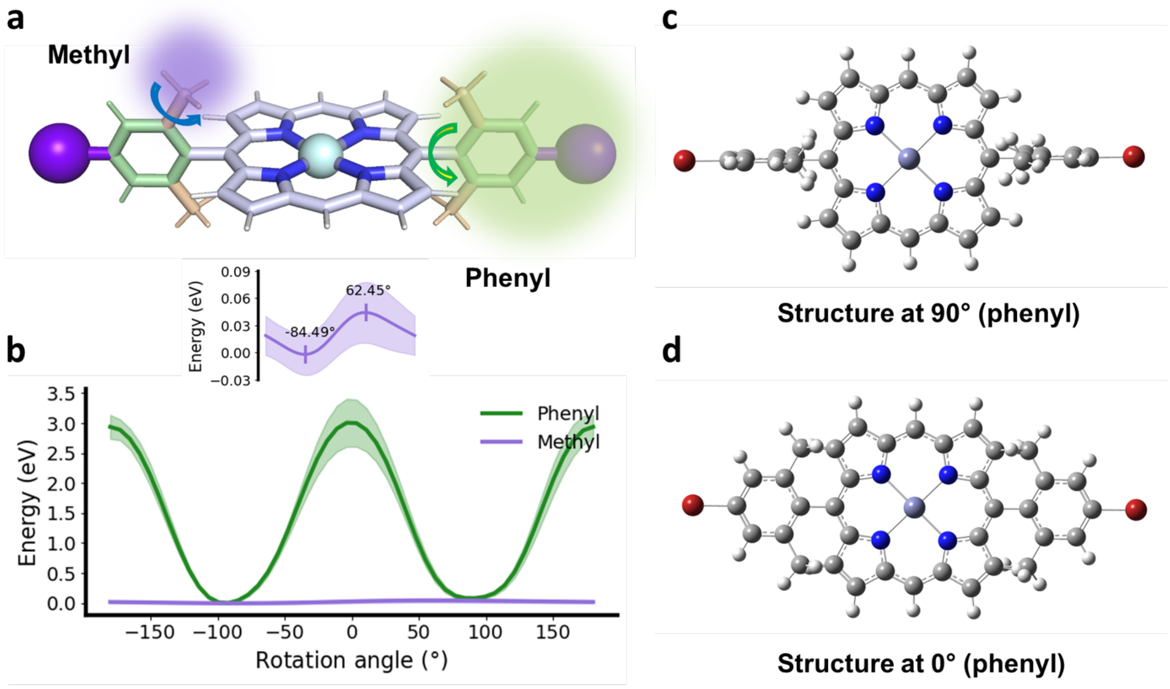

Figure S4: **Conformational analysis of $ZnBr_2Me_4DPP$ using BOSS.** (a) Schematic illustration of rotatable C(porphyrin)-C(phenyl) bond (purple shadow) and C(phenyl)-C(methyl) bond (green shadow). (b) Corresponding energy diagrams by scanning rotation angles. The inset magnifies the energy curve for the rotation around the C(phenyl)-C(methyl) bond. (c) Structure with the phenyl group at 90° angles around the C(porphyrin)-C(phenyl) bonds. (d) Structure with the phenyl group at 0° (Methyl groups in phenyls bump into the porphyrin ring).

The most important conformational change for the $ZnBr_2Me_4DPP$ molecule is due to rotation of the $C_6H_2(CH_3)_2Br$ (phenyl) groups relative to the central porphyrin. It should be noted that free rotation of the CH_3 (methyl) groups around the C-C bond that connect them to the phenyl is possible, but this rotation does not change the overall conformations of the porphyrin enough to warrant a separate analysis of the corresponding rotamers on the surface, as these minute structural changes are likely superseded by those induced by adsorbate-substrate interactions. The main conformer of $ZnBr_2Me_4DPP$ has the phenyl moieties orthogonal to the central porphyrin backbone, with free rotation being hindered by the methyl groups on the former (rotation barrier around 3.0 eV corresponding to clash between

atoms, see Fig. S4b). Futhermore, the backbone of the molecule has some limited flexibility, but since changes in this structure is most accurately described by collective motion of the molecule, it is difficult to assign simple variables required by BOSS, such as bond stretch, bending or torsion to this motion. Therefore, we also used the Conformer-Rotamer Ensemble Sampling Tool (CREST),[2] which employs metadynamics including collective variables to check for alternative gas phase conformers. However, this analysis also yielded one conformer only, in addition to a large number (around 300) of similar rotamers, corresponding to the aforementioned collective movement of the porphyrin backbone, the phenyl side groups, and the methyl groups.

Various configurations of $ZnBr_2Me_4DPP$ on Au(111)

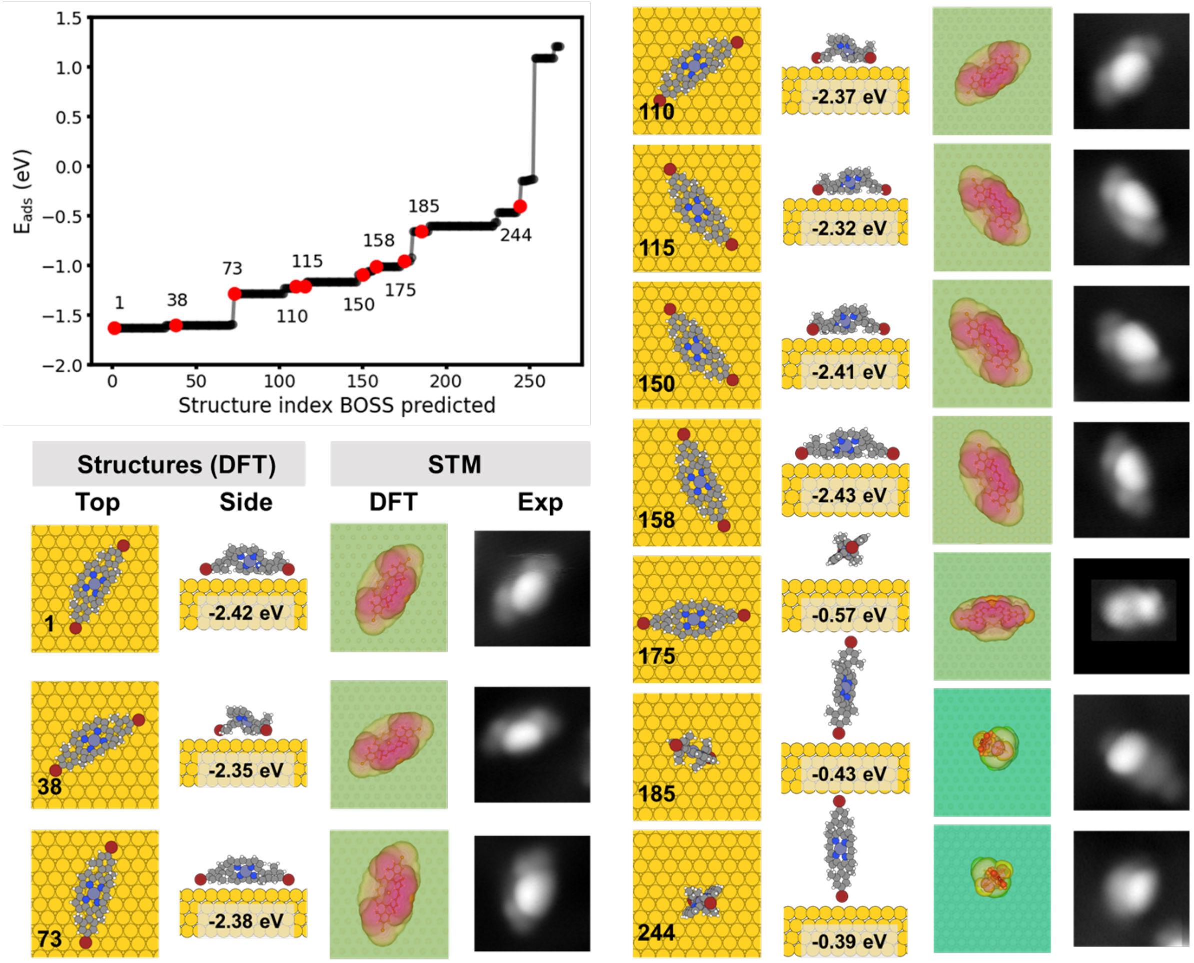

Figure S5: **Various configurations of $ZnBr_2Me_4DPP$ adsorbed on Au(111) searched by BOSS.** The energy diagram represents the searched structures and corresponding adsorption energies obtained from BOSS. Some stable configurations with possible corresponding STM scanning images are shown. From left to right, the columns represent 3D structures optimized by DFT calculations from top view and from side view, DFT-simulated STM, experimental scanning images (setpoint: 1×10^{-11} A, bias voltage: 1 V). The values on the side structures indicate the adsorption energies obtained by DFT calculations.

ResNet18 Block

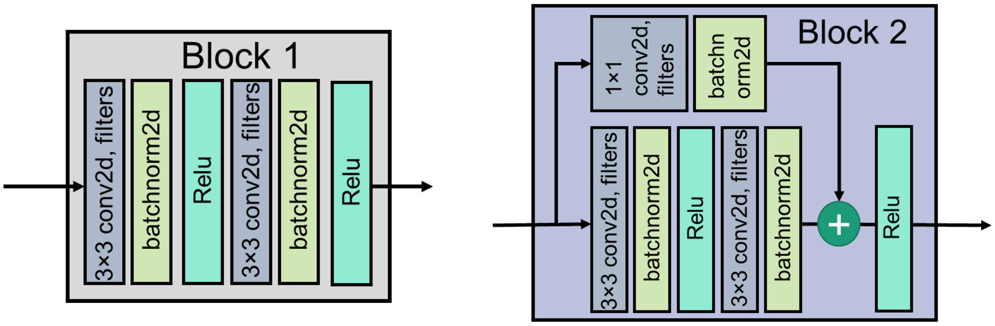

Figure S6: **Architectures of Block 1 and Block 2 in ResNet18.** Block 1 is stacked by two sets of convolution layer, normalization layer and Relu layer, which is a regular ResNet block. Block2 enhances a regular ResNet block by incorporating a 1×1 convolution with a normalization.

dI/dV spectra and maps of $ZnBr_2Me_4DPP$ on Au(111)

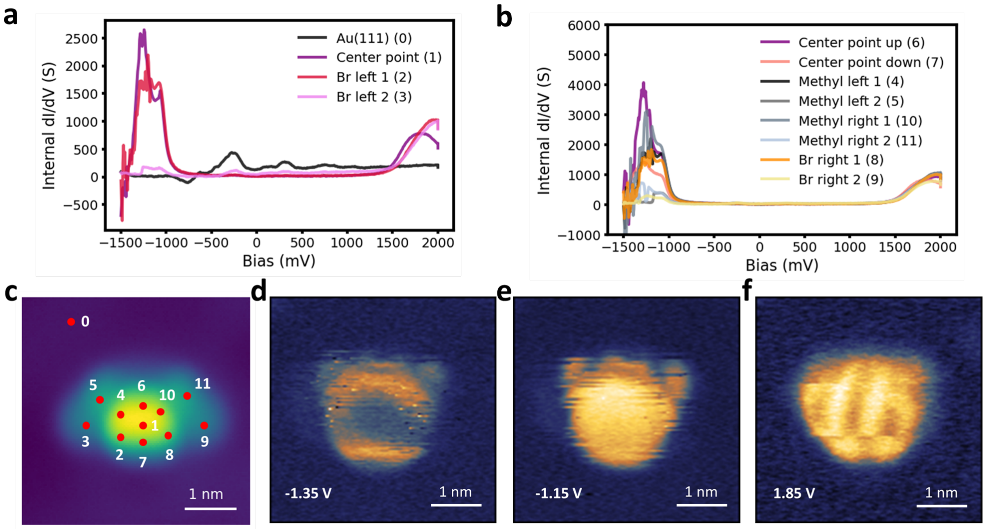

Figure S7: (a,b) Experimental dI/dV spectra at points 0 - 11 on the pattern corresponding to a Br_2Me_4DPP molecule in (c) an STM image (STS feedback loop at $5{\times}10^{-10}$ A and 2 V for all cases). (d, e,f) dI/dV maps recorded at -1.35 V (HOMO-1), -1.15 V (HOMO) and 1.85 V (LUMO), the setpoint is $5{\times}10^{-11}$ A, constant current mode.

Interpretation

Example of indeterminate cases

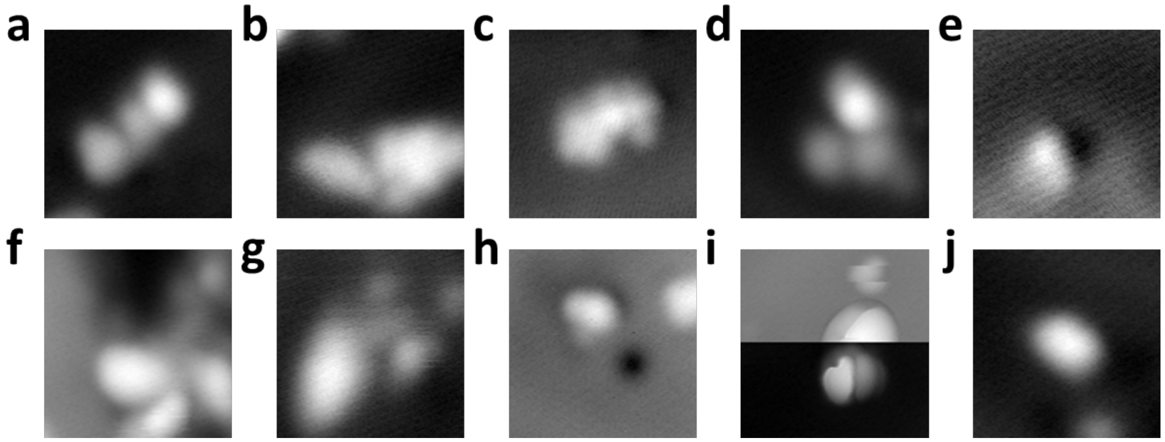

Figure S8: Example of indeterminate cases in STM images.

Properties of contrast patterns in STM images

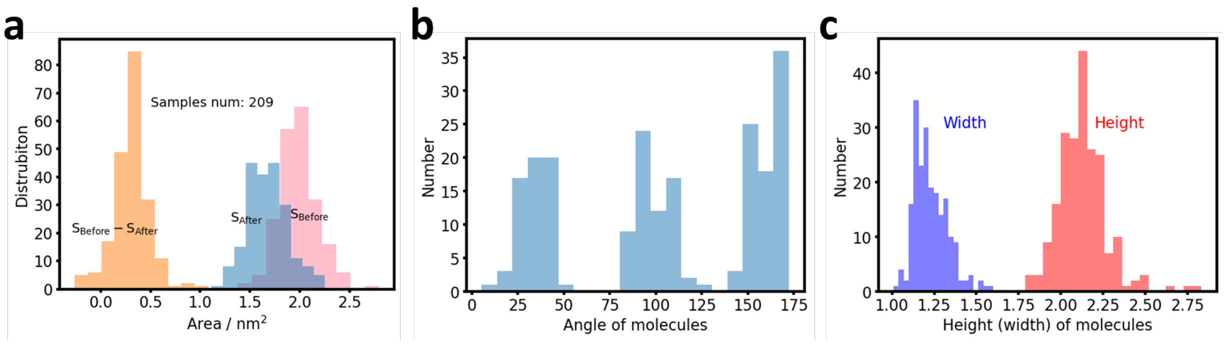

Figure S9: **Properties of contrast patterns in STM images.** (a) Distribution of areas of patterns of pristine $ZnBr_2Me_4DPP$ and their corresponding products ($ZnBrMe_4DPP^{\bullet}$ or $ZnMe_4DPP^{2\bullet}$) after implementing manipulations. (b) Distributions of angles, (c) heights and widths for the approximated ellipses from patterns of reactants $ZnBr_2Me_4DPP$. Data obtained by analysing 209 pairs of images among successful cases.

Reaction energies and possible dissociation mechanisms

In Fig. S10a, the STM images of pristine $ZnBr_2Me_4DPP$ (left), $ZnBrMe_4DPP^{\bullet}$ (center), $ZnMe_4DPP^{2\bullet}$ (right) are represented. Based on these, we explored the corresponding 3D structures of the $ZnBrMe_4DPP^{\bullet}$ or $ZnMe_4DPP^{2\bullet}$ products by DFT. When removing a Br atom from $ZnBr_2Me_4DPP$, the adjacent C atom in the $ZnBrMe_4DPP^{\bullet}$ product tends to bind with an atom in the Au(111) surface, simultaneously losing its initial radical character, as corroborated by spin-polarized DFT computations. Similar effects are observed after removing the second Br atom, and the two terminals of the $ZnMe_4DPP^{2\bullet}$ product bind strongly with the surface, the whole molecule adopting an "arc-type" conformation on the surface. After inspecting the z-coordinates of specific atoms before and after dissociation, we note that some atoms are elevated, while others are lowered with respect to their initial positions (Fig. S10c), which could be reflected in the experimental topography (Fig. 3a, red dot is the initial height of tip). Furthermore, the energy diagrams (Fig. S10e) obtained by DFT calculations for the removals of two bromines from $ZnBr_2Me_4DPP$ in both concurrent and consecutive models show that the activation barrier energy for the first Br removal (2.05 eV) is close to the second Br removal (1.82 eV), while a higher activation energy barrier of 3.66 eV needs to be surmounted for the concurrent removal of two bromines. Therefore, the dissociation of two bromine atoms from $ZnBr_2Me_4DPP$ could occur sequentially or simultaneously during the dissociation procedure.

To gain further insight on the possible reaction mechanism leading to the C-Br bond dissociation, we analyzed the electronic structure of $ZnBr_2Me_4DPP$. Highly localized C–Br antibonding orbitals were identified based on the feature in the PDOS corresponding to Br and C atoms at approximately 3.7 eV above the Fermi level (Fig. S10e). For the isolated gas-phase molecule, these correspond to LUMO+7 and LUMO+8. From visual inspection, the symmetries of the orbitals appear correct for a p-p σ^* bond, representing the antibonding counterpart for the single σ bond between the C and Br atoms. Based on the agreement with the most successful bias voltage determined by RDL at 3.9 V, and the energies of

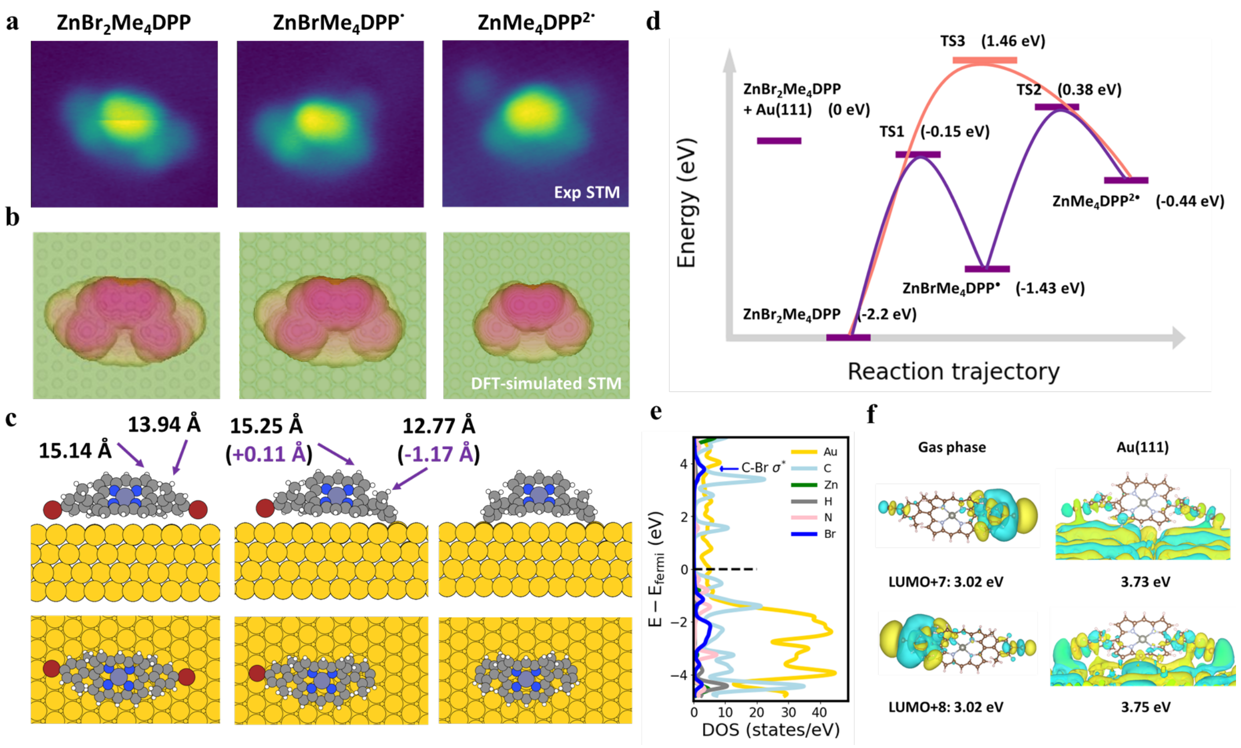

Figure S10: **Interpretation of reaction.** (a) STM images of $ZnBr_2Me_4DPP$, $ZnBrMe_4DPP^{\bullet}$ and $ZnMe_4DPP^{2\bullet}$. (b) Corresponding DFT-simulated STM images. (c) Corresponding 3D structures (top view and side view). (d) Corresponding energy diagrams from $ZnBr_2Me_4DPP$ to $ZnBrMe_4DPP^{\bullet}$ radical to $ZnMe_4DPP^{2\bullet}$ biradical. (e) Atom-Projected Density of States (PDOS) of $ZnBr_2Me_4DPP$ adsorbed on Au(111), all energies are relative to the Fermi level (-5.16 eV). The Au DOS are scaled by 0.05. (f) C–Br antibonding orbitals and states for the $ZnBr_2Me_4DPP$ molecule in the gas-phase as well as on surface.

the antibonding states, we surmise that one probable mechanism is dissociative electron attachment, where the tunneling electrons are supplied to the specific C–Br σ^* orbitals. Subsequently, the charge is neutralized, which is accompanied by vibrational excitation of the C–Br bond. Furthermore, this provides us with a way to rationalize the observation that in some instances, both C–Br bonds are dissociated during manipulation, as the antibonding states are delocalized over both C–Br bonds, in contrast to the gas-phase molecule, where one end is more prominent. Despite this consistent model, we cannot fully rule out other mechanisms, as we observe successful dissociation with a wide range of parameters and tip positions. Considering that the computed barriers for dissociation are significantly lower than the σ^* bond energies at 2.6 and 3.3 eV (Fig. S10e), respectively, another possible

mechanism proceeds via tunneling into orbitals of lower energy, followed by the vibrational excitation. The resulting excess vibrational energy can quickly be redistributed into the other modes, including the one corresponding to C–Br bond vibration. This is consistent with the more stochastic nature (lower rate of success) of the dissociation observed at these lower bias values, as sufficient vibrational energy needs to be partitioned into these specific bonds. Furthermore, this also rationalizes why higher bias values induce more reactions in general, as more energy becomes available for reactions.

Signal classifier

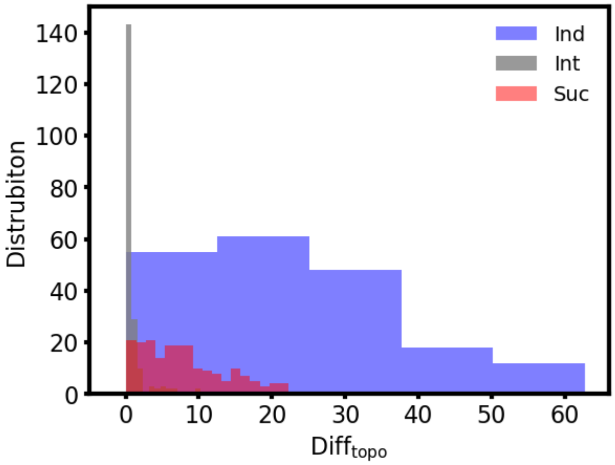

Figure S11: Distribution of $Diff_{topo}$ during dissociation for three categories of products. 200 samples were obtained by a random selection from each category.

Performance metrics of M_{Target}

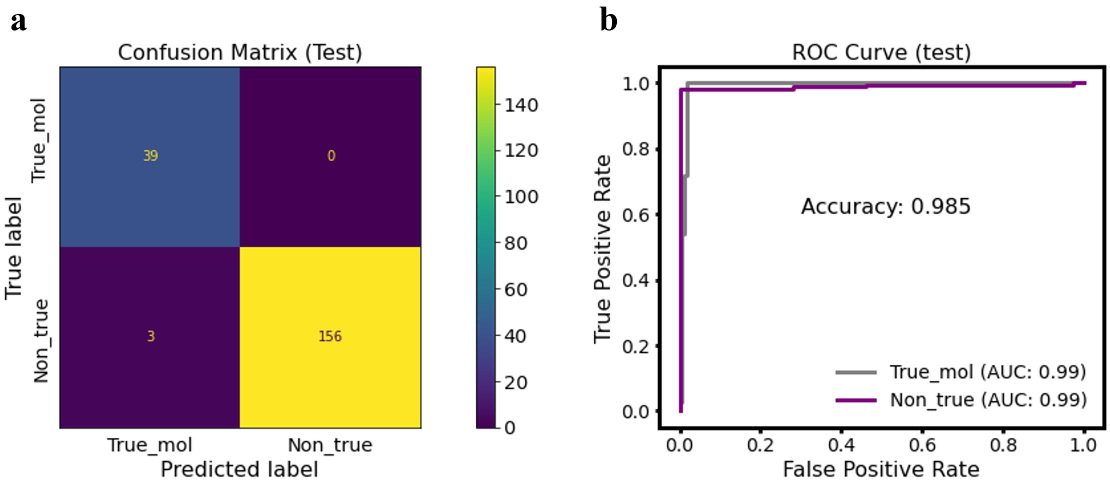

Figure S12: Performance metrics of M_{Target} for distinguishing $ZnBr_2Me_4DPP$ or not when detecting targeted reactants, with 1389 samples in the training dataset and 198 samples in the test dataset.

Table S1: Performance metrics of M_{Target}

class	precision	recall	f1-score	support
True_mol	0.93	1.00	0.96	39
Non_true	1.00	0.98	0.99	159
accuracy		0.98		198
macro avg	0.96	0.99	0.98	198
weighted avg	0.99	0.98	0.99	198

Performance metrics of M_{Triple}

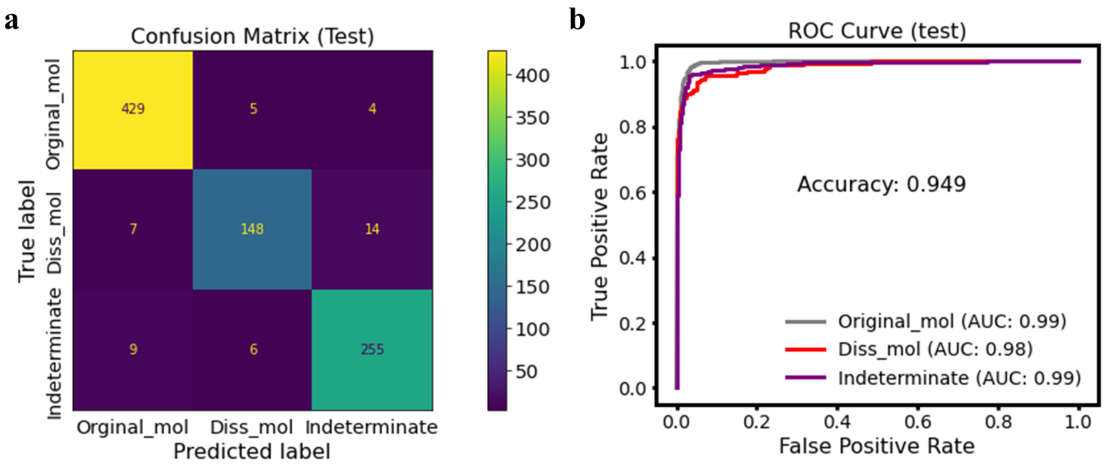

Figure S13: Performance metrics of M_{Triple} for classifying $ZnBr_2Me_4DPP$, dissociated molecules, indeterminate when evaluating products, with 3950 samples in the training dataset and 877 samples in the test dataset.

Table S2: Performance metrics of M_{Triple}

class	precision	recall	f1-score	support
Original_mol	0.96	0.98	0.97	438
Diss_mol	0.93	0.88	0.90	169
Indeterminate	0.93	0.94	0.94	270
accuracy		0.95		877
macro avg	0.94	0.93	0.94	877
weighted avg	0.95	0.95	0.95	877

Performance metrics of M_{Ind}

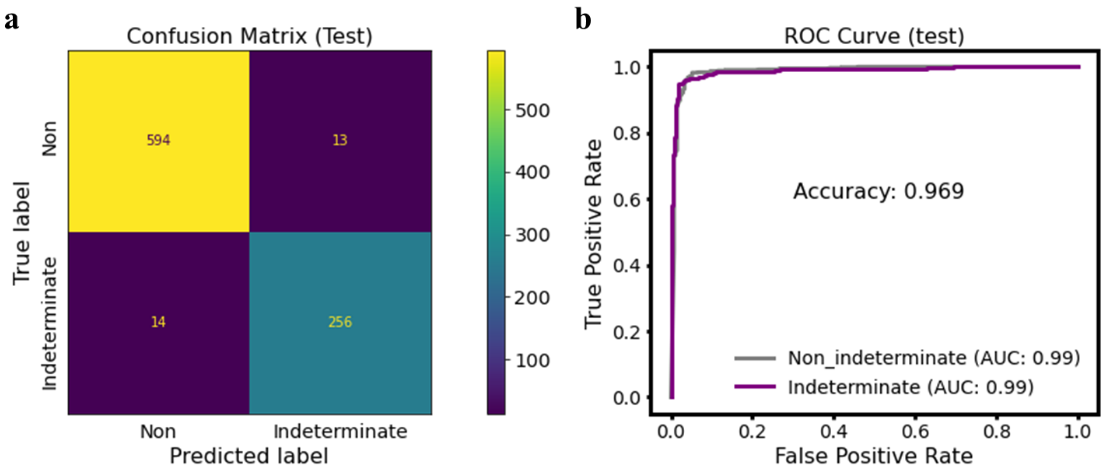

Figure S14: Performance metrics of M_{Ind} for distinguishing indeterminate products among products, with 3950 samples in the training dataset and 877 samples in the test dataset.

Table S3: Performance metrics of M_{Ind}

**class**	**precision**	**recall**	**f1-score**	**support**
Non_indeterminate	0.98	0.98	0.98	607
Indeterminate	0.95	0.95	0.95	270
accuracy		0.97		877
macro avg	0.96	0.96	0.96	877
weighted avg	0.97	0.97	0.97	877

Performance metrics of M_{Diss}

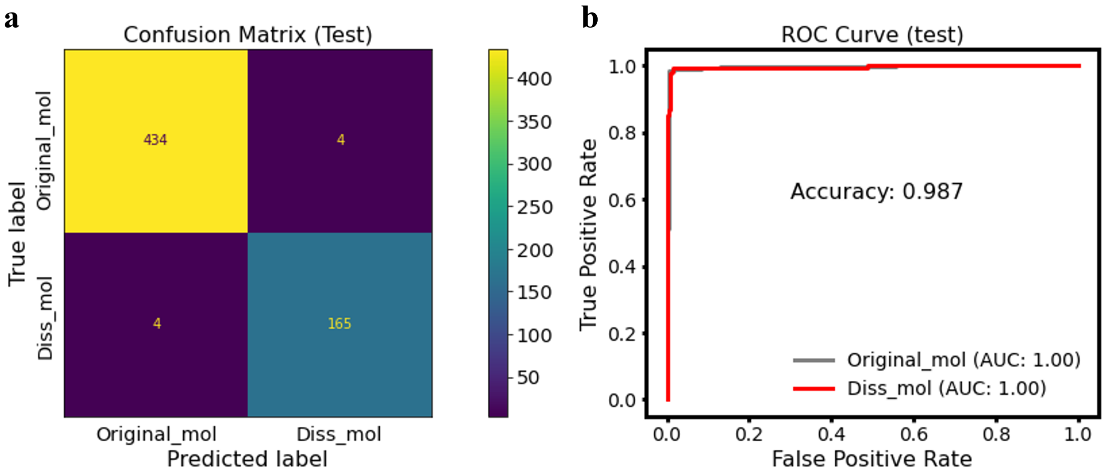

Figure S15: Performance metrics of M_{Diss} for distinguishing $ZnBr_2Me_4DPP$ and dissociated molecules when evaluating products, with 2764 samples in the training dataset and 607 samples in the test dataset.

Table S4: Performance metrics of M_{Diss}

class	precision	recall	f1-score	support
Original_mol	0.99	0.99	0.99	438
Diss_mol	0.98	0.98	0.98	169
accuracy		0.99		607
macro avg	0.98	0.98	0.98	607
weighted avg	0.99	0.99	0.99	607

Decision-making

Random action at different bias patterns

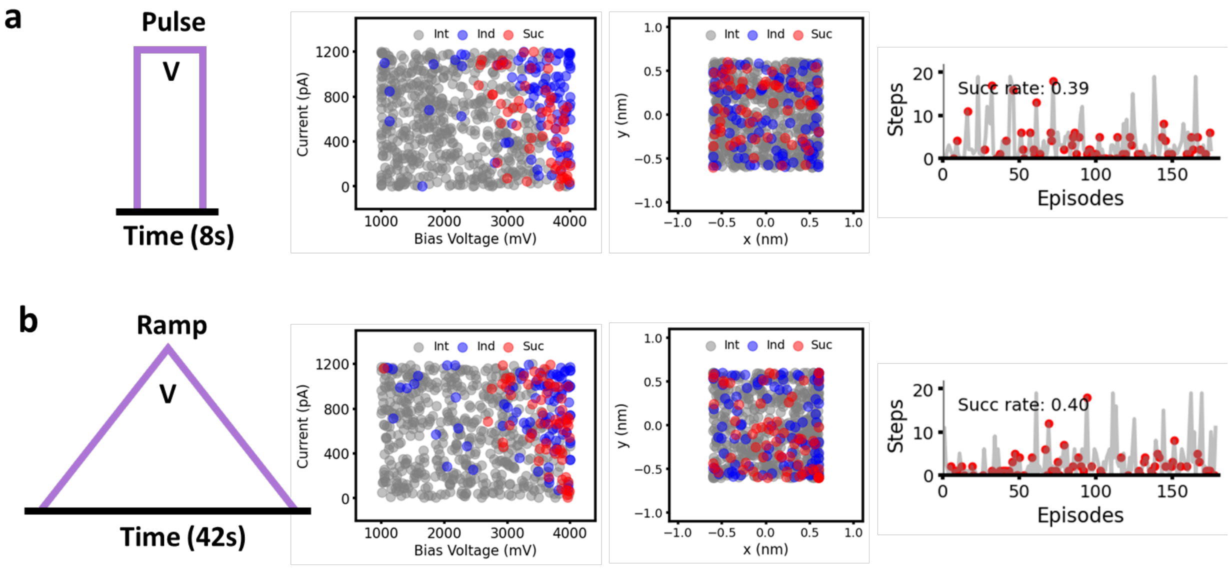

Figure S16: **Performance of randomly generating actions at two types of bias patterns with tip positions referred to the centre point of the ellipse constrained to within 0.6 nm along x (or y) axis.** (a) 8 s pulse pattern. (b) 42 s ramp pattern. The first column illustrates the bias voltage patterns, the second column indicates the distribution of Suc (red dots), Ind (blue dots), and Int species (grey dots) upon varying the current and the voltage, the third column shows the corresponding distribution upon varying the tip position (tip_x and tip_y), the fourth column represents the evolution of dissociation steps over multiple episodes, where red points symbolize successful dissociation.

Contrast pattern measurement

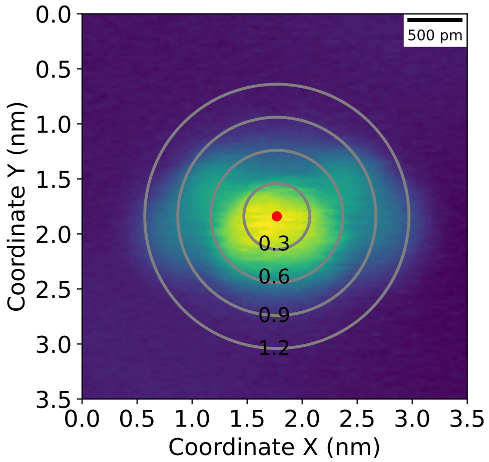

Figure S17: Schematic illustration of the distance of each part of $ZnBr_2Me_4DPP$ within a pattern approximated as an ellipse, referenced to its center point.

Random action with fixed tip position

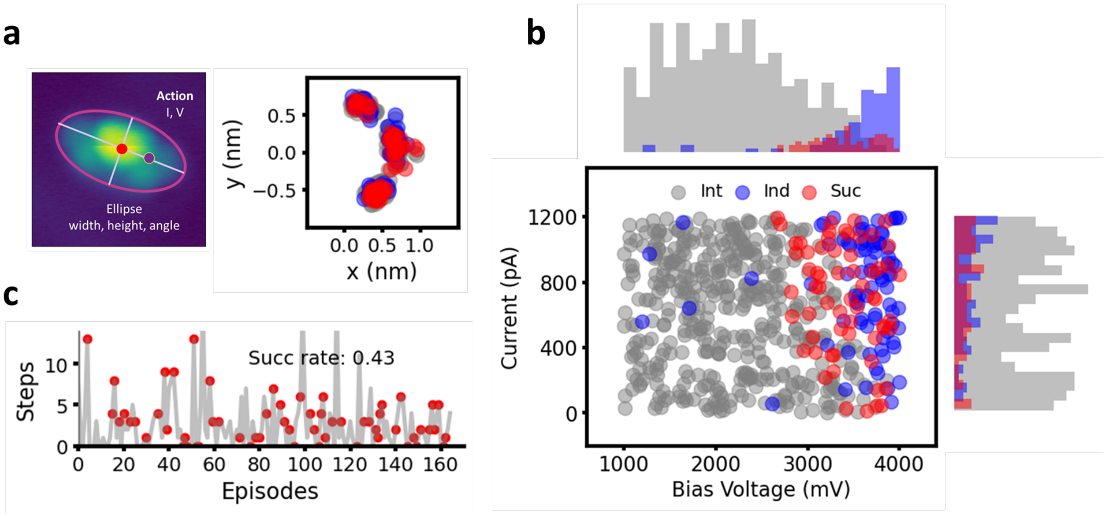

Figure S18: **Performance of random action at a fixed tip position.** (a) Left panel: the pattern corresponding to a molecule is approximated as an ellipse. Tip position referred to the center point of the ellipse. Right panel: the distribution of tip position in this test. (b) Distribution of voltages and currents. Three categories of products (Int, Ind, Suc) under the voltage-current pairs are marked by grey, red and blue. (c) The evolution of dissociation steps over multiple episodes, red points indicate successful dissociation. The specific tip position is set based on Equation 6.

Random selection from successful dissociation parameters

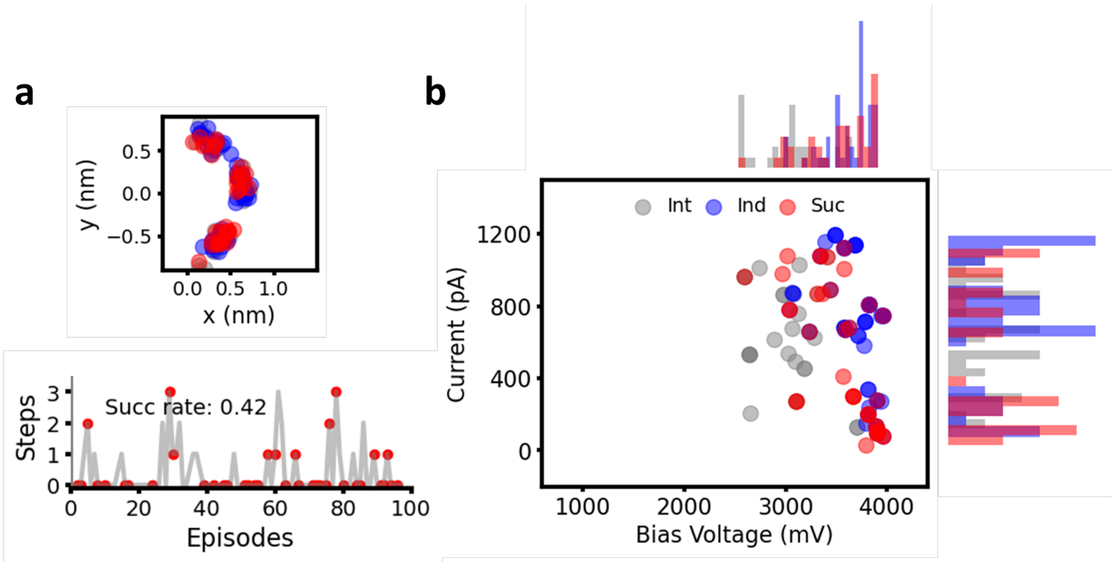

Figure S19: **Performance of randomly selecting actions from successful dissociation parameters.**(a) Upper panel: Distribution of the tip position, Lower pane: Evolution of dissociation steps over multiple episodes, red points indicate successful dissociation. (b) Distribution of voltages and currents.

Dissociation parameters from the DRL model

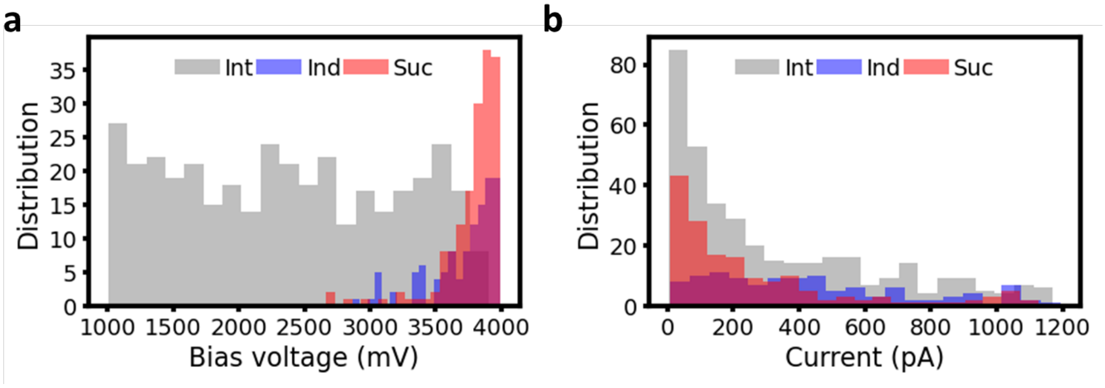

Figure S20: Distribution of bias voltages (a) and currents (b) under three categories of products (Int, Ind and Suc), marked by grey, red and blue, in the DRL training

Tip conditioning region

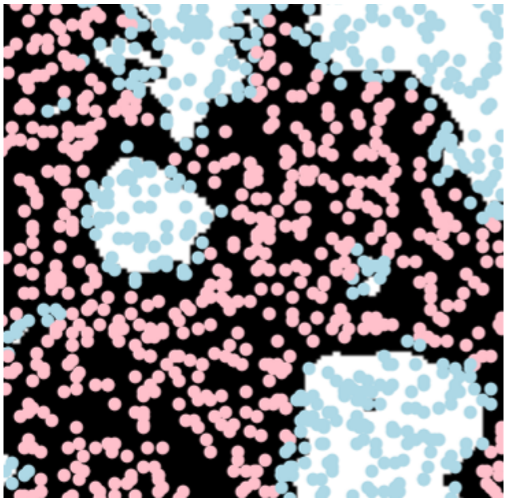

Figure S21: Schematic illustration of our algorithm detecting blank regions and selecting tip position from specific regions for conditioning tips.

Synthesis and characterization

Materials and Methods

Chemicals and solvents were purchased from commercial suppliers (Aldrich, BLDpharm, Thermo Scientific Chemicals and Scharlab) and used without further purification. All dry solvents were freshly distilled under argon over an appropriate drying agent before use. Column chromatography was carried out on silica gel VWR-60 (40-63 µm). Analytical TLC was performed on aluminum sheets precoated with silica gel 60 F-254 from Merck. ^{1}H- and ^{13}C-NMR spectra were recorded with a Bruker XRD-500 (500 MHz) instrument at room temperature (25 ºC). Chemical shifts values (δ) are referred to the corresponding deuterated solvent ($CDCl_3$). UV/vis experiments were carried out by using quartz cells with a 1 cm optical path length in a JASCO-V660 UV-vis spectrophotometer. MALDI-TOF MS spectra were obtained in a Bruker ULTRAFLEX III (MALDI-TOF/TOF) spectrometer.

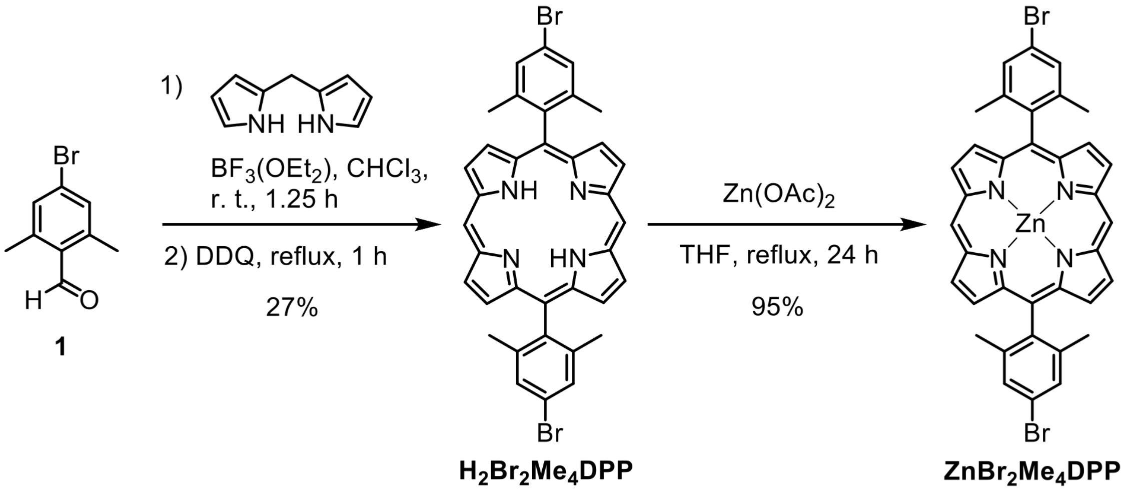

Figure S22: Synthetic route towards $ZnBr_2Me_4DPP$.

Synthesis of precursors $H_2Br_2Me_4DPP$

$H_2Br_2Me_4DPP$ was synthesized using a modified procedure from Zhao and co-workers.[3] meso-H-dipyrromethane (533 mg, 2.5 mmol, 1 eq.) and 2,6-dimethyl-4-bromobenzaldehyde (365

mg, 2.5 mmol, 1 eq.) were loaded in a flame-dried 500 mL round-bottom flask. $CHCl_3$ (250 mL) and dry ethanol (1.87 mL) were added to the flask and the mixture was vigorously degassed with argon for 40 minutes under stirring and in the dark. Then, $BF_3(OEt_2)$ (0.1 mL) was added under argon, whereupon the mixture turned dark red. The mixture was stirred in the dark for 80 minutes at room temperature, then DDQ (908 mg, 4 mmol, 1.6 eq.) was added in one batch and the mixture was heated to reflux for 1 h. After that, the mixture was allowed to cool down to room temperature, triethylamine (2 mL) was added and the solution stirred for 5 minutes. After this time, the solvent was removed under reduced pressure and the resulting crude purified by column chromatography using DCM/n-heptane (3:2) as eluent. From the column, a red-coloured fraction was isolated (Rf = 0.68) which upon removal of the solvent under reduced pressure afforded a solid which was suspended in n-pentane, sonicated, filtered, washed with n-pentane (4 mL), and dried under vacuum to yield $H_2Br_2Me_4DPP$ as a dark-violet powder (225 mg, 27%). ^{1}H-NMR (500 MHz, $CDCl_3$): δ = 10.26 (s, 2H), 9.37 (d, J = 4.6 Hz, 4H), 8.87 (d, J = 4.6 Hz, 4H), 7.69 (s, 4H), 1.86 (s, 12H), –3.10 (s, 2H); ^{13}C NMR (125 MHz, $CDCl_3$): δ = 142.0, 139.7, 132.4, 130.1, 129.9, 122.4, 115.9, 105.1, 21.7; MALDI-TOF MS (DCTB matrix): m/z (% intensity) = 674.1 – 681.1 m/z $[M]^+$ (100%); UV/vis ($CHCl_3$): λmax (log ϵ) = 405 (5.32), 500 (4.21), 576 (3.72).

^{1}H-NMR

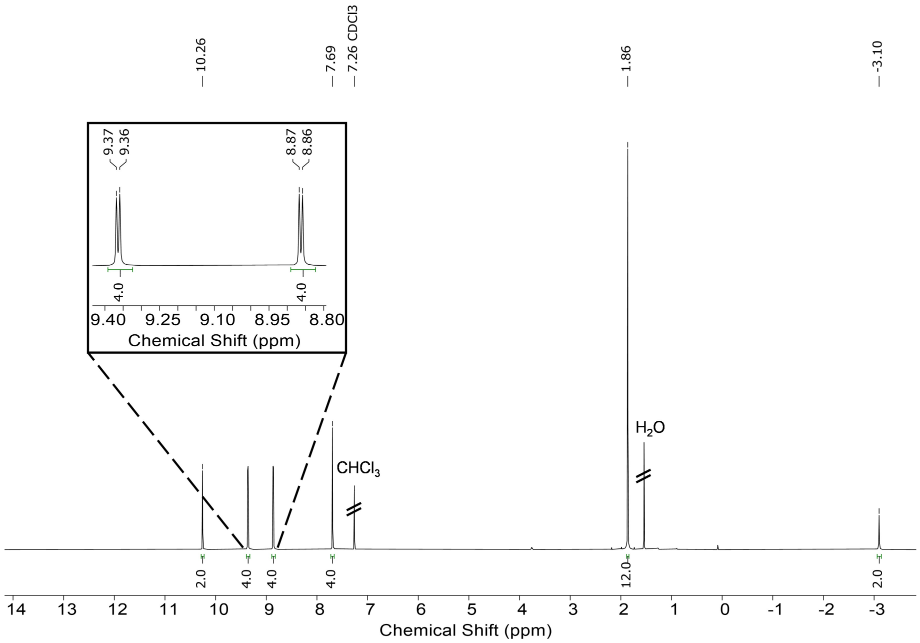

Figure S23: ^{1}H-NMR spectrum of $H_2Br_2Me_4DPP$ in $CDCl_3$ (⫽ = residual solvent signals).

^{13}C-NMR

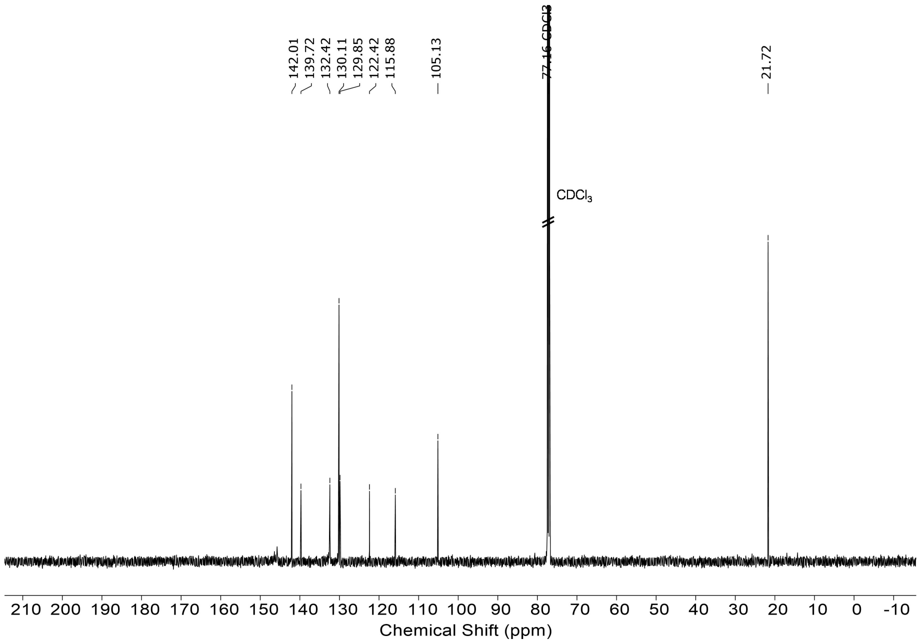

Figure S24: ^{13}C-NMR spectrum of $H_2Br_2Me_4DPP$ in $CDCl_3$ (⫽ = residual solvent signals).

MS

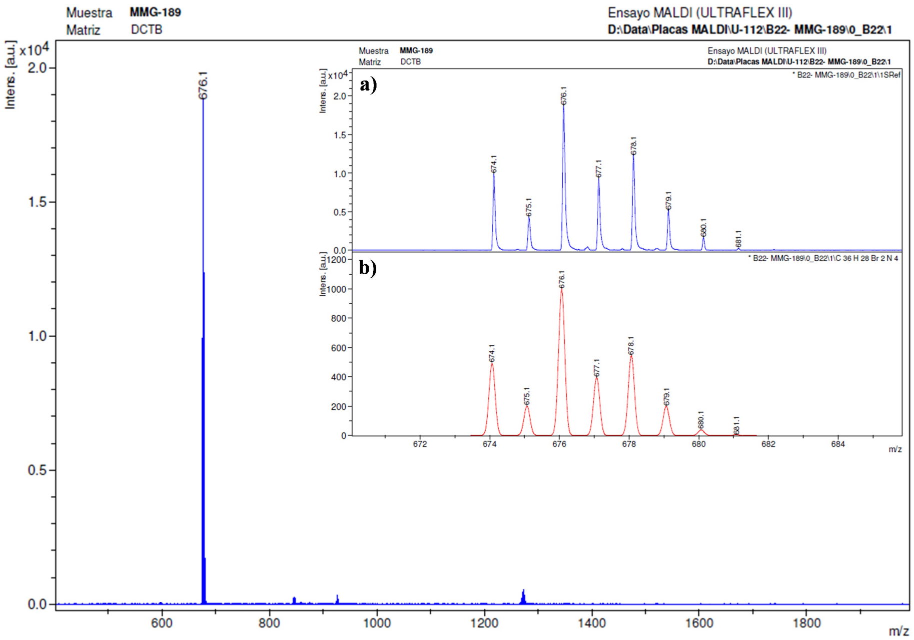

Figure S25: MALDI-TOF mass spectrum of $H_2Br_2Me_4DPP$. Inset: (a) experimental isotopic resolution of the MALDI-TOF main peak at 676.1 m/z. (b) Calculated isotopic pattern for $H_2Br_2Me_4DPP$.

UV/vis

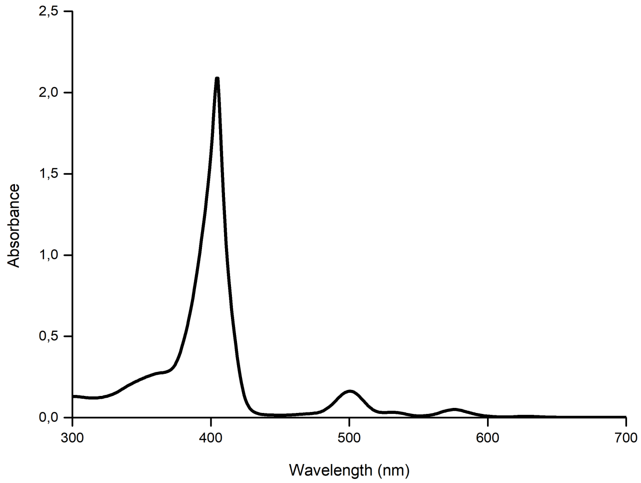

Figure S26: UV/vis spectrum of $H_2Br_2Me_4DPP$ in $CHCl_3$ (conc. = 10 µM).

Synthesis of $ZnBr_2Me_4DPP$

$H_2Br_2Me_4DPP$ (20 mg, 0.03 mmol) and $Zn(OAc)_2$ (54 mg, 0.30 mmol, 10 eq.) were loaded in a flame-dried 10 mL Schlenk tube and three cycles of vacuum/argon backfilling were applied. Then, dry THF (5 mL) was added, and the mixture was heated to reflux while stirring under argon for 24 h. After this time, the mixture was then allowed to cool to room temperature, quenched with water (10 mL) and extracted with DCM (3 × 15 mL). The combined organic layers were washed with water (25 mL) and brine (25 mL), dried over anhydrous $MgSO_4$, filtered and the filtrate dried under reduced pressure. The resulting crude was subjected to a short column chromatography using DCM/n-heptane (3:2) as eluent, isolating a red-coloured fraction (Rf = 0.61) which solvent was removed under reduced pressure. The resulting solid was suspended in n-pentane, sonicated, filtered, washed with n-pentane (3 × 2 mL), and dried under vacuum to yield $ZnBr_2Me_4DPP$ as a purple powder (21 mg, 95%). ^{1}H-NMR (500 MHz, $CDCl_3$): δ = 10.28 (s, 2H), 9.42 (d, J = 4.5 Hz, 4H), 8.94 (d, J = 4.5 Hz, 4H), 7.68 (s, 4H), 1.83 (s, 12H); ^{13}C-NMR (125 MHz, $CDCl_3$): δ = 149.8, 149.5, 141.9, 140.9, 132.8, 131.1, 129.9, 122.1, 116.9, 106.1, 21.7; MALDI-TOF MS (DCTB matrix): m/z (% intensity) = 736.0 – 746.0 m/z $[M]^+$ (100%); UV/vis ($CHCl_3$): λmax (log ϵ) = 410 (5.34), 540 (4.22).

^{1}H-NMR

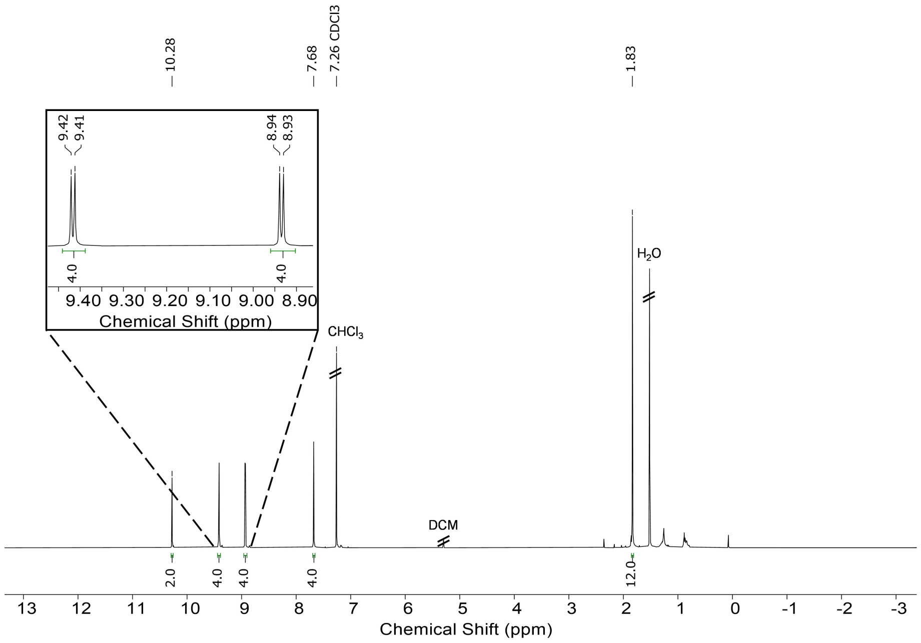

Figure S27: ^{1}H-NMR spectrum of $ZnBr_2Me_4DPP$ in $CDCl_3$ (⁄⁄ = residual solvent signals).

^{13}C-NMR

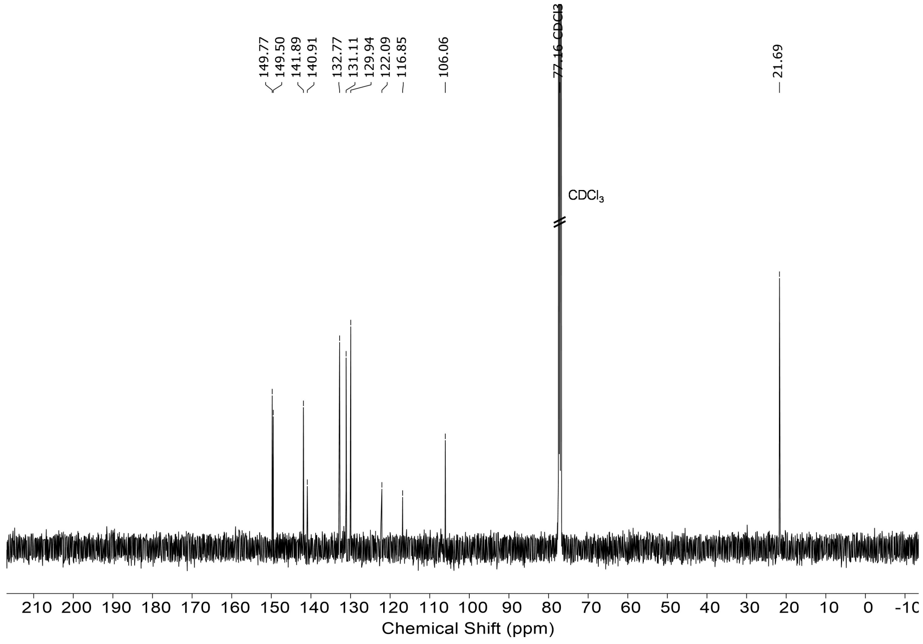

Figure S28: ^{13}C-NMR spectrum of $ZnBr_2Me_4DPP$ in $CDCl_3$ (⁄⁄ = residual solvent signals).

MS

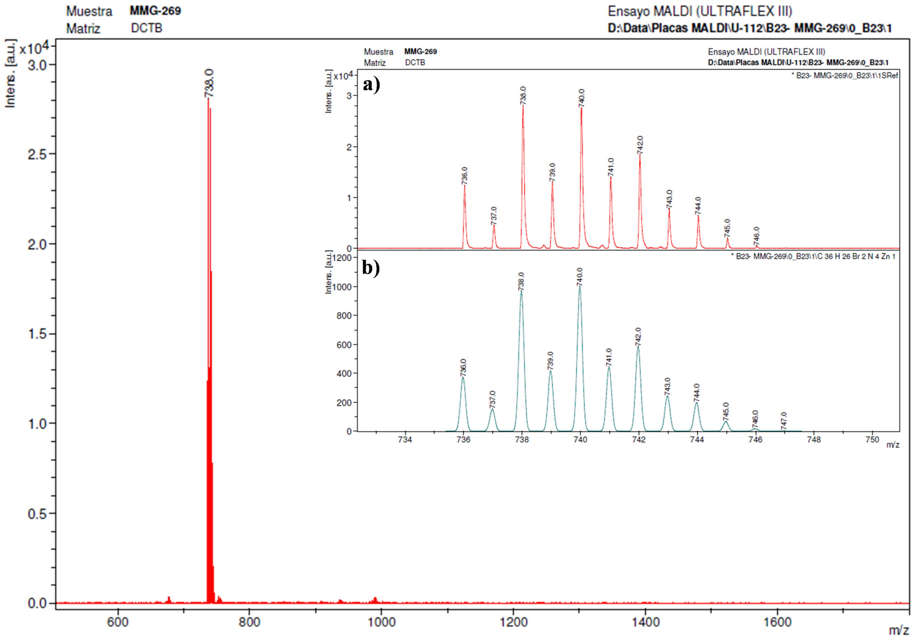

Figure S29: MALDI-TOF mass spectrum of $ZnBr_2Me_4DPP$. Inset: (a) experimental isotopic resolution of the MALDI-TOF main peak at 738.0 m/z. (b) Calculated isotopic pattern for $ZnBr_2Me_4DPP$.

UV/vis

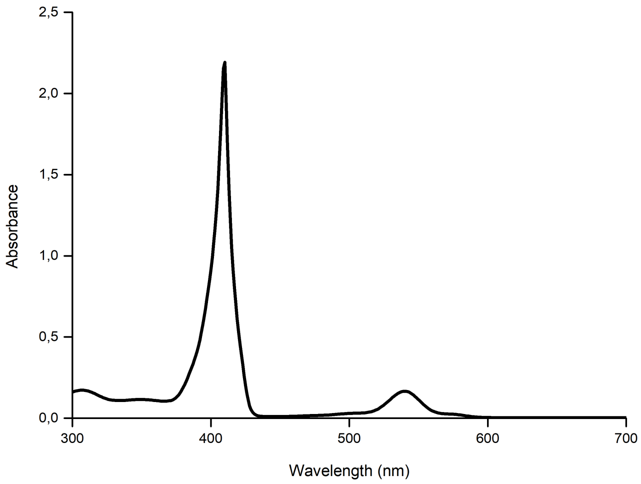

Figure S30: UV/vis spectrum of $ZnBr_2Me_4DPP$ in $CHCl_3$ (conc. = 10 µM).

References

(1) Edmondson, M.; Saywell, A. Molecular Diffusion and Self-Assembly: Quantifying the Influence of Substrate hcp and fcc Atomic Stacking. *Nano Lett.* **2022**, *22*, 8210–8215.

(2) Pracht, P.; Bohle, F.; Grimme, S. Automated exploration of the low-energy chemical space with fast quantum chemical methods. *Phys. Chem. Chem. Phys.* **2020**, *22*, 7169–7192.

(3) Zhao, Y.; Jiang, K.; Li, C.; Liu, Y.; Zhu, G.; Pizzochero, M.; Kaxiras, E.; Guan, D.; Li, Y.; Zheng, H.; Liu, C.; Jia, J.; Qin, M.; Zhuang, X.; Wang, S. Quantum nanomagnets in on-surface metal-free porphyrin chains. *Nat. Chem.* **2023**, *15*, 53–60.
